# Hybrid modeling of electroporation and impedance spectroscopy for label free characterization of stem cells

**DOI:** 10.1038/s41598-026-62691-0

**Published:** 2026-07-23

**Authors:** Sameh Sherif, Yehya H. Ghallab, Yehea Ismail

**Affiliations:** 1 School of Electronics, Communications and Computer Engineering, Egypt-Japan University of Science and Technology (E-JUST), New Borg El Arab, Egypt; 2 Biomedical Engineering Department, Capital University (Formerly Helwan University), Cairo, Egypt; 3 Center of Nanoelectronics and Devices (CND), Zewail City of Science and Technology, The American University in Cairo (AUC), Giza, Egypt

**Keywords:** Electroporation, Transmembrane potential, Pore density kinetics, Stem-cell characterization, Pulsed electric field, Membrane conductivity, Microfluidic electroporation platform, Numerical modeling, Impedance spectroscopy, Label-free classification, Biological techniques, Biophysics, Biotechnology, Cell biology, Engineering

## Abstract

Label-free, non-destructive characterization of stem-cell differentiation states remains an important goal in regenerative medicine and cell therapy. Existing computational frameworks commonly treat electroporation either at the tissue scale or for simplified single-cell geometries, and relatively few studies connect time-domain electroporation observables with swept-frequency impedance features measured in a microfluidic platform. This study presents a revised hybrid analytical–numerical and experimental framework for comparing undifferentiated human mesenchymal stem cells (hMSCs) with osteogenic-committed hMSCs. The numerical models are parameterized using the cell-type values : an undifferentiated hMSC model with representative radius $R_{\textrm{U}}={10}\,\upmu \hbox {m}$, cytoplasmic conductivity $\sigma _{i,\textrm{U}}={0.32}\,\hbox {S m}^{-1}$, membrane capacitance $C_{m,\textrm{U}}=1\times 10^{-2}\,\hbox {F m}^{-2}$, and characteristic electroporation voltage $U_{\textrm{ep,U}}={0.258}\,\text {V}$; and an osteogenic hMSC model with $R_{\textrm{O}}={13}\,\upmu \hbox {m}$, $\sigma _{i,\textrm{O}}={0.24}\,\hbox {S m}^{-1}$, $C_{m,\textrm{O}}=8\times 10^{-3}\,\hbox {F m}^{-2}$, and $U_{\textrm{ep,O}}={0.32}\,\text {V}$. Both models are placed in the same microfluidic electrode environment and excited by electric-field pulses (${1}\,\hbox {kV cm}^{-1}$ to ${5}\,\hbox {kV cm}^{-1}$, rise time 1 ns). The passive Schwan RC time constants are ${4.69\times 10^{-7}}\,\text {s}$ for undifferentiated hMSCs and ${5.96\times 10^{-7}}\,\text {s}$ for osteogenic hMSCs; the plotted post-threshold rise times are shorter, on the order of ${1\times 10^{-7}}\,\text {s}$ to ${2\times 10^{-7}}\,\text {s}$. The passive polar transmembrane potentials at ${5}\,\hbox {kV cm}^{-1}$ are approximately ${7.5}\,\text {V}$ and 9.75 V, respectively. Swept-frequency impedance spectroscopy (${1}\,\hbox {kHz}$ to ${1}\,\hbox {MHz}$) performed on undifferentiated and osteogenic-committed hMSCs provides the matched frequency-domain comparison: low-frequency impedance, series resistance, reactance trough depth, phase angle, and voltage-dependent impedance drop are extracted at applied voltages of 1, 5, 10, 15, 20, and 25 V. The experimental data show that osteogenic hMSCs have higher baseline impedance ($|Z|={11029}\,\Omega$ vs. ${7863}\,\Omega$ at ${1}\,\text {V}$, ${1}\,\hbox {kHz}$), whereas undifferentiated hMSCs exhibit the stronger high-voltage impedance drop at ${25}\,\text {V}$ approximately (92.4 % compared with 86.9 % for osteogenic hMSCs). Calibrated 10.4 *μ*m and 24.9 *μ*m polystyrene microbeads are included as cell-free size standards for the impedance workflow. The combined results define a cell-type feature space ${\mathcal {F}}_{\textrm{combined}} = \{\tau _{\textrm{charge}},\; V_m,\; N(t),\; r_p(t),\; \sigma _m(t),\; f_c,\; \Delta |Z|_{f_{c,0}},\; R_{s,1\,\textrm{kHz}},\; |X_{s,\textrm{pk}}|\}$ for future label-free classification studies. The present work should be interpreted as a matched modelling and impedance-analysis framework; definitive biological classification, direct pore imaging, viability validation, and trained classifier performance remain outside the scope of this study.

## Introduction

Electroporation the transient permeabilization of lipid-bilayer membranes by externally applied electric fields has become a cornerstone technology in modern biomedicine. When a cell is exposed to an electric pulse whose amplitude and duration exceed membrane-specific thresholds, hydrophilic pores nucleate across the lipid bilayer, sharply increasing membrane permeability to ions, drug molecules, plasmid DNA, and other membrane-impermeant cargo^1,2^. The process is commonly described through three coupled stages: (i) capacitive charging of the membrane, (ii) stochastic pore nucleation once the transmembrane potential $V_m$ approaches a critical range (typically ${0.2}\,\text {V}$ to ${1}\,\text {V}$), and (iii) pore expansion followed by partial or complete resealing after the pulse, as captured by dynamic pore-density and pore-radius models^3,4^. The highest pore densities concentrate at the depolarized and hyperpolarized poles of a spherical cell facing the field direction, whereas the largest pore radii can occur near the borders of electroporated regions^4,5^.

The efficacy and selectivity of electroporation are governed by a confluence of factors: the amplitude, duration, and repetition rate of the applied field; the biophysical properties of the cell (membrane capacitance, cytoplasmic conductivity, cell radius); and physical variables of the surrounding medium such as temperature and ionic strength^6,7^. In particular, the electric field amplitude required for cellular electroporation typically falls in the range of ${1\times 10^{3}}\,\hbox {V cm}^{-1}$ to ${1\times 10^{4}}\,\hbox {V cm}^{-1}$, scaling inversely with cell size owing to the transmembrane potential’s linear dependence on cell radius^8^. Upon field application, charge accumulates on both leaflets of the membrane, generating $V_m$; the magnitude of $V_m$ directly determines the rate of pore nucleation, and once $V_m$ exceeds the threshold voltage $V_{\textrm{ep}}$, the pore creation rate accelerates exponentially, enabling mass transport between the intracellular and extracellular compartments^9^.

Nanosecond pulsed electric fields (nsPEFs) have attracted considerable recent interest because they can perturb not only the plasma membrane but also intracellular membranes while limiting bulk Joule heating under appropriately selected pulse conditions^5,9,10^. In stem-cell systems, nsPEFs have also been reported to modulate differentiation potential and functional behavior, underscoring the need to distinguish reversible biophysical permeabilization from longer-term biological responses^11–13^. Optimizing electroporation protocols for stem-cell delivery and characterization therefore requires a quantitative, physics-based understanding of how pulse parameters interact with cell size, membrane capacitance, membrane composition, and medium conductivity.

Prior computational efforts have elucidated fundamental electroporation mechanisms in single-cell and tissue-scale systems^3–5,7^ and have characterized medium-conductivity effects on thresholds^6^. In parallel, impedance-based methods have been used for label-free monitoring of stem-cell differentiation and single-cell biophysical analysis^14–18^, while impedance and light-scattering methods have also been demonstrated for leukemic-cell discrimination^19–22^. However, three critical gaps persist. First, no existing framework simultaneously resolves all four electroporation observables (transmembrane potential, pore density, pore radius, and dynamic conductivity) within a microfluidic geometry tailored to stem cells. Second, pore-radius kinetics, which govern expansion and resealing after nucleation, have rarely been connected explicitly to an impedance-spectroscopy interpretation workflow. Third, quantitative features beyond $V_m$ and *N* that may discriminate stem cells of different differentiation states under identical pulse conditions remain insufficiently defined and compared with measured impedance spectra. This work addresses all three gaps through four specific contributions:

1. A hybrid analytical–numerical model coupling Laplace’s equation for quasi-static electric potential with both a nonlinear Smoluchowski-type pore-density evolution equation and a pore-radius kinetic ordinary differential equation, implemented within a finite-element time-dependent solver.
2. Parametric application of the model to two experimentally relevant hMSC states using representative radii selected from reported MSC size distributions and osteogenic-differentiation morphology studies: undifferentiated hMSCs represented by $R_{\textrm{U}}={10}\,\upmu \hbox {m}$ and osteogenic-committed hMSCs represented by $R_{\textrm{O}}={13}\,\upmu \hbox {m}$^15,17,23^ in a realistically dimensioned microfluidic channel with coplanar electrodes.
3. Evaluation of membrane charging time, passive transmembrane-potential scaling, pore-density kinetics, pore-radius evolution, and membrane-conductivity changes as mechanistic descriptors, with explicit distinction between simulation-derived state variables and experimentally measured impedance features.
4. Construction of a nine-dimensional combined feature space ${\mathcal {F}}_{\textrm{combined}}$ integrating time-domain electroporation features with frequency-domain impedance spectroscopy features, evaluated alongside swept-frequency measurements on undifferentiated and osteogenic-committed hMSCs as a quantitative basis for future label-free stem-cell classification studies.

The remainder of this paper is organized as follows. “Methods” presents the theoretical model, numerical implementation, and microfluidic impedance-spectroscopy framework. “Results and discussion” presents the simulation results and quantitative model–data comparison. “Comparative analysis” provides comparative analysis with the literature. “Impedance-based experimental characterization of stem cells under electroporation” presents the impedance-based experimental characterization of hMSCs. Conclusions and limitations are given in “Conclusion”.

## Methods

This section details the theoretical foundation, numerical implementation, and microfluidic experimental framework used to study single-cell electroporation dynamics and swept-frequency impedance spectroscopy. To ensure reproducibility and clarity, a Traceability Table (Table 1) is provided, mapping every figure, table, and reported dataset to its data-generation source.

**Table 1:** Traceability table mapping figs, tables, and reported datasets to their primary generation source and methodology.

Fig. / table	Data type	Primary source	Methodology & Context
Fig. 1	Schematic	CAD layout	Device dimensions and polar segments
Fig. 2	Schematic	Analytical model	Equivalent-circuit single-shell model
Table 2	Equations	Theory	Governing mathematical relations
Table 1	Metadata	Review	Revision traceability summary
Table 3	Parameters	Literature/experiment	Input constants for COMSOL model
Table 4	Calibration	Impedance analyzer	Calibrated |*Z*| values for 10.4 *μ*m and 24.9 *μ*m microbeads at 10 kHz, 100 kHz, and 1 MHz
Figs. 5, 6	Simulation	COMSOL FEM	$V_m(t)$ transient polar charging
Figs. 7, 8	Simulation	COMSOL FEM	Pore density *N*(*t*) evolution
Figs. 9, 10	Simulation	COMSOL FEM	Pore radius $r_p(t)$ evolution
Figs. 11, 12	Simulation	COMSOL FEM	Spatial profile of $\sigma _m$ vs. arc length
Figs. 13, 14	Simulation	COMSOL FEM	Post-pulse relaxation & resealing
Table 5	Simulation results	COMSOL FEM	Extracted parameters ($\tau _{\textrm{charge}}$, $V_{\textrm{clamp}}$)
Table 6	Simulation comparison	COMSOL FEM	Numerical percent comparison between undifferentiated and osteogenic hMSC models
Fig. 3	Experimental image	Laboratory photograph	Photograph of impedance test bench
Fig. 4	Experimental image	Microscopy	Fabricated coplanar electro-focusing array
Fig. 15	Experimental	Impedance analyzer	Swept-frequency |*Z*| vs. frequency
Table 9	Experimental results	Impedance Analyzer	Extracted crossover and impedance drops
Table 7	Processed data	Feature extraction	Percentage impedance drop vs. voltage
Table 8	Experimental	Impedance analyzer	Series resistance $R_s$ vs. frequency
Table 10	Processed data	Feature extraction	Tabulated series parameters and contrast

### Theoretical modeling

#### Cell geometry and single-shell representation

Each stem cell is approximated as a spherical single-shell model comprising two concentric homogeneous dielectric regions: the cytoplasm (interior) and the surrounding lipid-bilayer membrane of thickness $d_m$. In this representation, the cell is electrically equivalent to a passive resistive–capacitive (RC) circuit in which the membrane capacitance $C_m = \varepsilon _m / d_m$ (per unit area) and the membrane conductance $G_m = \sigma _m / d_m$ (per unit area) govern the transient charging response to an applied electric field. This single-shell idealization captures the essential physics of transmembrane polarization while remaining computationally tractable for matched comparison of the two experimentally studied hMSC populations.

**Fig. 1:**
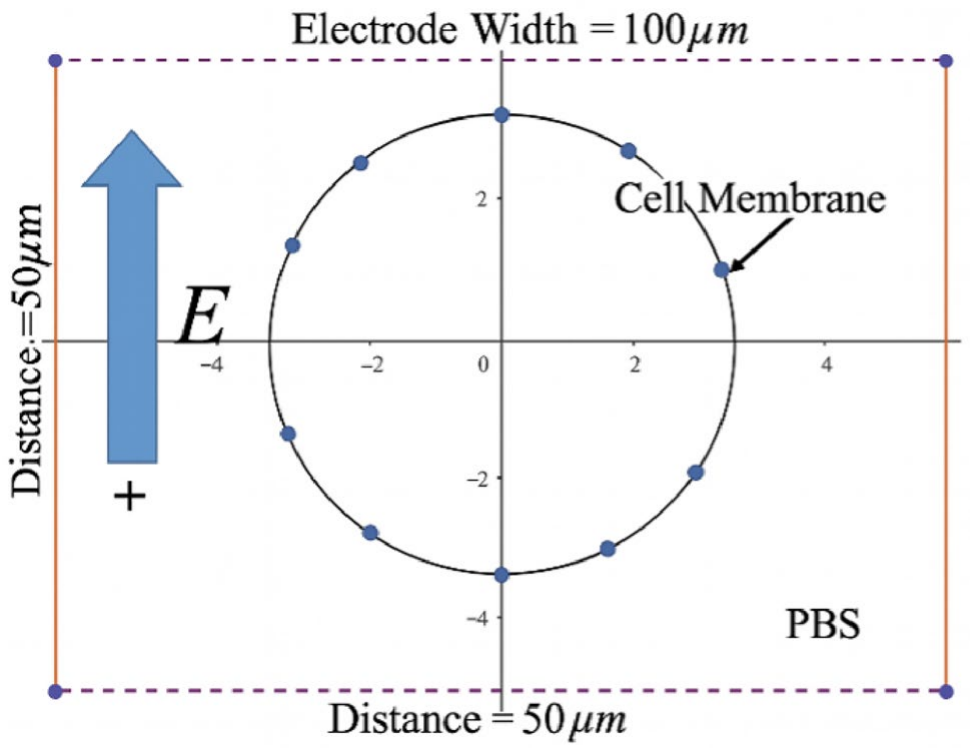
Schematic of the proposed microfluidic electroporation platform used for both hMSC populations. The undifferentiated hMSC model uses $R_{\textrm{U}}={10}\,\upmu \hbox {m}$ and the osteogenic hMSC model uses $R_{\textrm{O}}={13}\,\upmu \hbox {m}$. The numerical parameter set uses PBS conductivity $\sigma _e={0.32}\,\hbox {S m}^{-1}$ and a representative electrode separation of ${50}\,\upmu \hbox {m}$ for field estimation, while the impedance spectra are reported by the externally applied voltages used in the experiments. The external electric field ${\bf E}$ induces transmembrane polarization, with the hyperpolarized pole (H) and depolarized pole (D) experiencing the largest potential magnitudes. Four principal membrane poles and two border regions are identified for segmented analysis.

**Fig. 2:**
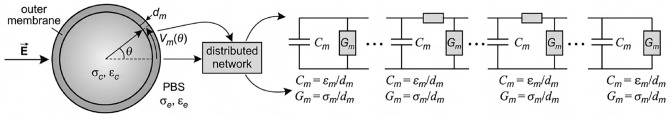
Electrical equivalent-circuit representation of the spherical single-shell stem-cell model. The cell membrane of thickness $d_m$ is represented as a distributed parallel RC network with capacitance $C_m = \varepsilon _m/d_m$ and conductance $G_m = \sigma _m/d_m$ per unit area. The cytoplasmic interior is characterized by conductivity $\sigma _c$ and permittivity $\varepsilon _c$, while the extracellular PBS medium is characterized by $\sigma _e$ and $\varepsilon _e$. The angle *θ* is measured from the direction of the applied electric field ${\bf E}$ and determines the local transmembrane potential through the $\cos \theta$ dependence of Eq. (2).

Two cell-type models are considered. The undifferentiated hMSC model is assigned a representative radius $R_{\textrm{U}} = {10}\,\upmu \hbox {m}$, while the osteogenic-committed hMSC model is assigned $R_{\textrm{O}} = {13}\,\upmu \hbox {m}$ to reflect the larger apparent cell size and altered electrical phenotype after osteogenic differentiation. These values are representative modelling radii selected from reported MSC size distributions and from impedance/morphology studies showing differentiation-associated changes in hMSC electrical and morphological properties^15,17,23^; they are not intended to imply a universal fixed radius for every cell. The cytoplasmic conductivity and membrane capacitance are also cell-type specific, rather than being forced to a single shared value: $\sigma _{i,\textrm{U}}={0.32}\,\hbox {S m}^{-1}$ and $C_{m,\textrm{U}}=1\times 10^{-2}\,\hbox {F m}^{-2}$ for undifferentiated hMSCs, compared with $\sigma _{i,\textrm{O}}={0.24}\,\hbox {S m}^{-1}$ and $C_{m,\textrm{O}}=8\times 10^{-3}\,\hbox {F m}^{-2}$ for osteogenic hMSCs. The computational model includes the extracellular medium (PBS) and a homogeneous cytoplasmic interior; the plasma membrane is treated as an internal boundary condition rather than a volumetric domain, consistent with the thin-shell approximation^3,5^. A separate nuclear subdomain is not meshed in this single-shell implementation; the nuclear parameters from the previous publication are therefore documented as reference values but not used as independent fitted variables in the present impedance comparison. A schematic of the cell geometry and its orientation with respect to the applied electric field is shown in Fig. 1, while the electrical equivalent-circuit representation is depicted in Fig. 2.

For comprehensive analysis, each cell membrane is segmented into four principal poles and two border regions between each pair of adjacent poles. This segmentation strategy increases the spatial resolution of the region of interest, enabling a more thorough evaluation of the angular distribution of electrical parameters across the cell surface and yielding detailed, pole-resolved simulation results.

#### Governing equation for electric potential distribution

The electric potential distribution in each subdomain of the computational model is obtained by solving the quasi-static form of Maxwell’s equations. In the absence of free charges, the governing equation reduces to the time-dependent charge-conservation (continuity) equation^5^:

1 $$\begin{aligned} -\nabla \cdot \!\bigl (\sigma _i\,\nabla V\bigr ) -\nabla \cdot \frac{\partial \bigl (\varepsilon _i\,\nabla V\bigr )}{\partial t} = 0\;, \end{aligned}$$

where *V* denotes the electric potential, and $\varepsilon _i$ and $\sigma _i$ are the permittivity and conductivity, respectively, of subdomain *i*. In the present single-shell implementation, the index *i* runs over the extracellular medium and the homogeneous cytoplasmic interior. Equation (1) is solved numerically using the Electric Currents application mode of the AC/DC module in a time-dependent study solver, thereby capturing both conduction and displacement current contributions throughout the transient charging process.

#### Transmembrane potential formulation

For a spherical cell endowed with a passive RC membrane and subjected to a uniform external electric field, the induced transmembrane potential $V_m$ at angular position *θ* (measured from the field direction) is given by the classical expression^3,8^:

2 $$\begin{aligned} V_m = \frac{3\,E\,R\,\cos \theta }{2}\;, \end{aligned}$$

where *E* is the applied electric field strength and *R* is the cell radius. Equation (2) reveals two essential dependencies: $V_m$ scales linearly with both *E* and *R*, implying that larger cells reach the electroporation threshold at lower field strengths; and the $\cos \theta$ factor ensures that the maximum transmembrane potential magnitudes occur at the hyperpolarized pole ($\theta = \pi$) and the depolarized pole (*θ = 0*), while the equatorial regions ($\theta = \pi /2$) experience negligible polarization.

#### Current density through the membrane boundary

Because the cell membrane is treated as a thin internal boundary rather than a volumetric domain, the current density ${\bf J}$ traversing the membrane is modeled using a distributed impedance boundary condition^5^:

3 $$\begin{aligned} {\bf n}\cdot {\bf J} = \frac{\sigma _m}{d_m}\bigl (V - V_{\textrm{ref}}\bigr ) + \frac{\varepsilon _m}{d_m} \biggl (\frac{\partial V}{\partial t} -\frac{\partial V_{\textrm{ref}}}{\partial t}\biggr ), \end{aligned}$$

where ${\bf n}$ is the outward-pointing surface unit normal vector, *V* is the potential on the cytoplasmic side of the membrane, $V_{\textrm{ref}}$ is the reference potential on the extracellular side, $\varepsilon _m$ is the membrane permittivity, $\sigma _m$ is the membrane conductivity, and $d_m$ is the membrane thickness. The first term on the right-hand side of Eq. (3) represents the conductive (ohmic) current through the membrane, while the second term captures the displacement (capacitive) current. Together, these terms encode the RC character of the lipid bilayer and govern the transient charging dynamics observed in the transmembrane potential waveforms.

#### Pore density evolution kinetics

The nucleation and proliferation of hydrophilic pores on the membrane are described by a first-order nonlinear ordinary differential equation in which the pore density *N* depends on the instantaneous transmembrane potential^3,5^:

4 $$\begin{aligned} \frac{\partial N}{\partial t} = \alpha \,\exp \!\biggl [\biggl (\frac{V_{\textrm{TM}}}{V_{\textrm{ep}}} \biggr )^{\!2}\biggr ] \left[ 1 - \frac{N}{N_0}\, \exp \!\biggl (-\,q\biggl (\frac{V_{\textrm{TM}}}{V_{\textrm{ep}}} \biggr )^{\!2}\biggr )\right] , \end{aligned}$$

where $N_0$ is the equilibrium pore density in the absence of a transmembrane potential (i.e., the non-electroporated state), $V_{\textrm{ep}}$ is the characteristic electroporation voltage, *α* is the pore creation rate coefficient, *q* is a dimensionless electroporation parameter, and $V_{\textrm{TM}}$ denotes the local transmembrane potential. The exponential dependence on $(V_{\textrm{TM}}/V_{\textrm{ep}})^2$ ensures that pore density increases sharply once the transmembrane potential exceeds $V_{\textrm{ep}}$, consistent with the experimentally observed threshold behavior of electroporation. The bracketed factor $[1 - (N/N_0)\exp (-q(V_{\textrm{TM}}/V_{\textrm{ep}})^2)]$ provides a self-limiting mechanism: as *N* grows, the pore creation rate diminishes, preventing unphysical divergence.

#### Pore radius evolution kinetics

Once pores have nucleated, their radii evolve on a slower time scale governed by the balance between the electric driving force (which tends to expand pores) and the line tension of the lipid bilayer (which tends to close them). Following the formulation of Krassowska and Filev^4^, the time evolution of the radius $r_p$ of a single representative pore is described by:

5 $$\begin{aligned} \frac{dr_p}{dt} = \frac{D_p}{k_BT} \left[ \frac{2\pi \gamma }{r_p} - \frac{\pi \sigma _{\textrm{tens}}r_p^2}{r_p} + \frac{\pi \beta }{r_p^3} + \frac{F_E(V_{\textrm{TM}},r_p)}{2\pi r_p}\right] , \end{aligned}$$

where $D_p$ is the pore diffusion coefficient, $k_BT$ is the thermal energy, *γ* is the membrane edge tension, $\sigma _{\textrm{tens}}$ is the membrane surface tension, *β* is the steric repulsion energy coefficient, and $F_E(V_{\textrm{TM}},r_p)$ is the electrostatic energy term that depends on the local transmembrane potential and the pore geometry. In the present implementation, a simplified algebraic form consistent with the DeBruin–Krassowska model is employed: the pore radius at each membrane segment is updated at each time step using the instantaneous pore density *N* and the local transmembrane potential, ensuring self-consistent coupling between Eqs. (4) and (5). This kinetic description captures both pore expansion during the pulse phase and the gradual pore contraction during membrane resealing after pulse cessation.

The analytical framework of this study is constructed upon the mathematical features and governing equations summarized in Table 2.

**Table 2:** Mathematical features and governing equations employed in the hybrid analytical–numerical model. Each feature is linked to its role within the computational framework.

Feature / Parameter	Symbol / equation	Description	Role in model
Electric potential distribution	Eq. (1)	Governing equation for electrical potential in each subdomain	Solved in AC/DC time-dependent module for spatiotemporal potential fields
Transmembrane potential	Eq. (2): $V_m= 3ER\cos \theta /2$	Steady-state reference voltage across the membrane induced by the applied field	Determines the electroporation threshold reference
Current density	Eq. (3)	Conduction and displacement current across the membrane	Defines distributed impedance boundary condition
Pore density evolution	Eq. (4)	Time-dependent pore creation based on membrane potential	Models electroporation kinetics as function of time and angle
Pore radius evolution	Eq. (5)	Kinetic equation for pore expansion and resealing	Coupled to pore density; governs pore size dynamics and conductivity
Cell model type	Single-shell RC circuit	Equivalent representation of the membrane	Analytical reference for the FEM time-domain solution
Stimulus function	pulse (rise = 1 ns)	Nanosecond electric field excitation	Represents the applied electroporation pulse while limiting simulated Joule heating
Electric field range	$1\times 10^3$–$5\times 10^3$ V/cm	Applied external field magnitude used in the simulations	Determines transition between non-permeabilized and permeabilized states
Permittivity ($\varepsilon _i$)	Domain-dependent	Dielectric property of each region	Used for displacement current computation
Conductivity ($\sigma _i$)	Domain-dependent	Electrical conductivity of each subdomain	Defines steady-state current and influences charging time constant

### Numerical implementation

#### Simulation platform and solver configuration

All simulations are performed using the Electric Currents application mode within the AC/DC module of COMSOL Multiphysics, configured for a time-dependent study. The governing equation (1) is discretized on an unstructured triangular mesh with adaptive refinement concentrated at the membrane boundary, where the steepest potential gradients occur. To ensure mesh independence, a grid convergence analysis was conducted by systematically refining the mesh until the maximum transmembrane potential and pore density varied by less than *0.1%*. The final optimized mesh contains 45,210 elements, with an average element size of $0.05\ \upmu \text {m}$ along the cell membrane boundary.

The coupled system of the potential equation and the pore-density ordinary differential equation (4) is advanced in time using an implicit backward-differentiation-formula (BDF) solver with adaptive time stepping. The solver’s relative tolerance is set to $10^{-3}$, and the minimum time step is limited to 0.1 ns to accurately resolve the sub-nanosecond rise time of the excitation pulse.

To ensure complete reproducibility and clarify the modeling workflow, we explicitly distinguish the primary computational solver from the supplementary code. The quantitative electroporation dynamics, spatial distributions, and extracted parameters reported in this manuscript (including Figs. 5, 6, 7, 8, 9, 10, 11, 12, 13, 14 and Table 5) were generated exclusively using the high-fidelity finite-element method (FEM) solver in COMSOL Multiphysics, as it solves the fully coupled electrical-conduction and membrane-permeabilization problem across the segmented 2D domain. The supplementary Python script (electroporation_simulation.py) provided in the supplementary information acts solely as a standalone, illustrative implementation of a simplified single-point coupled ODE system. It uses a modified Schwan-type analytical approximation for a spherical cell to estimate the transmembrane potential and calculates pore density and radius kinetics at a single polar site. This script is intended for demonstration and quick parameter sweeps; it does not solve the spatial PDE domain and was not used to generate any of the primary figs or numerical features reported in the main text.

#### Boundary conditions and domain discretization

The computational domain comprises the microchannel region filled with PBS using the numerical parameter-set conductivity ($\sigma _e = {0.32}\,\hbox {S m}^{-1}$, $\varepsilon _r = 80$) and the cytoplasmic interior of the cell. In accordance with the single-shell approximation, the cell interior is modeled as a single homogeneous domain representing the cytoplasm, and a separate nuclear subdomain is not explicitly defined or meshed. The plasma membrane is implemented as a distributed impedance boundary condition according to Eq. (3), thereby avoiding the computational expense of volumetrically meshing the nanometer-thick lipid bilayer. The source electrode is assigned a time-dependent potential corresponding to the applied pulse, and the ground electrode is held at zero potential. The remaining boundaries of the microchannel are assigned electrically insulating (zero normal current density) conditions.

Initial conditions are set as *V(t=0)=0* throughout the domain and $N(t=0) = N_0$ (equilibrium pore density) on the membrane boundary. The membrane is segmented into four principal poles and two border regions, as described in “Cell geometry and single-shell representation”, and all electroporation observables ($V_m$, *N*, pore radius $r_p$, and membrane conductivity $\sigma _m$) are recorded at each segment as functions of time.

#### Algorithmic framework

The complete computational procedure is formalized in Algorithm 1, which details the initialization, electric-field application, time-stepped computation, and feature extraction stages. The algorithm accepts as inputs the microfluidic system configuration, cell biophysical properties, and excitation parameters, and returns the time-resolved electroporation observables together with the extracted feature set ${\mathcal {F}} = \{\tau _{\textrm{charge}},\; V_{\textrm{clamp}},\; N_{\max },\; r_{p,\max },\; \sigma _{m,\max }\}$.

**Algorithm 1:**
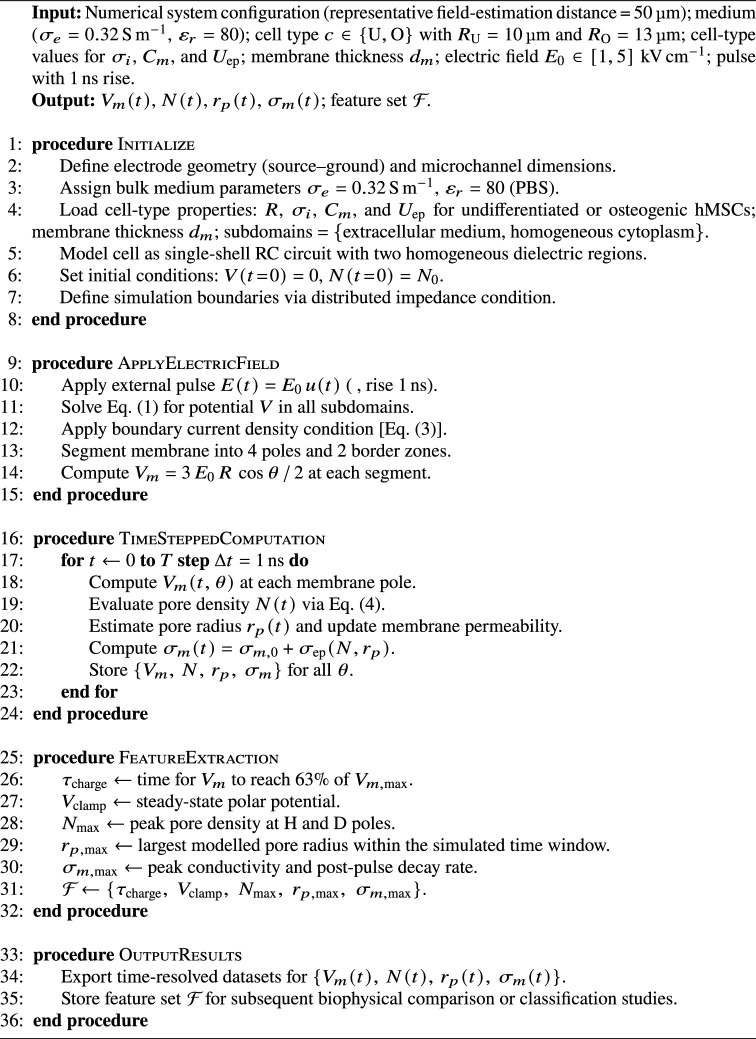
Hybrid analytical–numerical electroporation feature extraction for undifferentiatedand osteogenic hMSCs.

#### Simulation parameters and physical consistency

The excitation signal is a step function with a rise time of 1 ns, producing electric field amplitudes in the range ${1}\,\hbox {kV cm}^{-1}$ to ${5}\,\hbox {kV cm}^{-1}$ within the modelled microchannel. The use of nanosecond-scale pulses ensures that thermal effects remain minimal: the brief pulse duration results in negligible temperature increases in the sample, as established in prior studies^10,24^. The two numerical cases are therefore treated as cell-type-matched simulations: undifferentiated hMSCs are represented by $R_{\textrm{U}}={10}\,\upmu \hbox {m}$ and osteogenic hMSCs by $R_{\textrm{O}}={13}\,\upmu \hbox {m}$, with cell-type-specific cytoplasmic conductivity, capacitance, and electroporation threshold voltage. The representative field-estimation distance used in the previous parameter set is ${50}\,\upmu \hbox {m}$; the impedance experiments are reported by applied voltage to avoid overstating a one-to-one field equivalence across geometries. The total simulation time extends to ${1}\,\upmu \hbox {s}$, sufficient to capture membrane charging, pore formation, and the initial phase of membrane resealing.

The relationship between the analytical Schwan-type formula (which assumes a uniform electric field and spherical geometry) and the numerical FEM model is clarified: while the Schwan equation is useful for initial parameter estimation in a uniform field, the FEM model captures the spatial non-uniformity of the electric field arising from the coplanar electrode configuration in the microchannel. This non-uniformity is particularly critical for cells located close to the electrodes, where the electric field gradient is steepest.

Table 3 lists the physical, electrical, and kinetic parameter values used in the numerical simulations. These values represent established biophysical parameters for mammalian cells under electroporation conditions.

**Table 3:** Physical, electrical, and kinetic parameter values used in the numerical simulations.

Parameter description	Symbol	Value	Reference / source
Cell-type representative radii	$R_{\textrm{U}}$, $R_{\textrm{O}}$	${10}\,\upmu \hbox {m}$, ${13}\,\upmu \hbox {m}$	Representative modelling radii based on reported MSC size distributions and osteogenic hMSC morphology/electrical studies^15,17,23^
Membrane thickness	$d_m$	5 nm	^3^
Extracellular medium conductivity (PBS)	$\sigma _e$	${0.32}\,\hbox {S m}^{-1}$	Previous-publication parameter set
Cytoplasmic conductivity	$\sigma _{i,\textrm{U}}$, $\sigma _{i,\textrm{O}}$	${0.32}\,\hbox {S m}^{-1}$, ${0.24}\,\hbox {S m}^{-1}$	Undifferentiated / osteogenic hMSCs
Specific membrane capacitance	$C_{m,\textrm{U}}$, $C_{m,\textrm{O}}$	$1\times 10^{-2}\,\hbox {F m}^{-2}$, $8\times 10^{-3}\,\hbox {F m}^{-2}$	Undifferentiated / osteogenic hMSCs
Initial membrane conductance	$G_m$	${100}\hbox {S m}^{-2}$	Previous-publication parameter set
Initial membrane conductivity	$\sigma _{m,0}=G_m d_m$	${5\times 10^{-7}}\,\hbox {S m}^{-1}$	Derived from $G_m$ and $d_m$
Extracellular medium relative permittivity	$\varepsilon _e$	80	Standard
Cytoplasm relative permittivity	$\varepsilon _c$	80	^6^
Membrane relative permittivity	$\varepsilon _m$	$C_m d_m/\varepsilon _0$	Derived from cell-type $C_m$
Initial pore density	$N_0$	${1.5 \times 10^{9}}\,\hbox {m}^{-2}$	^3^
Pore creation rate coefficient	*α*	${1\times 10^{9}}\,\hbox {m}^{-2}\,\hbox {s}^{-1}$	^3^
Characteristic electroporation voltage	$U_{\textrm{ep,U}}$, $U_{\textrm{ep,O}}$	${0.258}\,\text {V}$, ${0.32}\,\text {V}$	Undifferentiated / osteogenic hMSCs
Dimensionless electroporation parameter	*q*	2.46	^5^
Pore diffusion coefficient	$D_p$	${5\times 10^{-14}}\hbox {m}^{2}\,\hbox {s}^{-1}$	Previous-publication parameter set
Minimum pore radius	$r_{p,\min }$	0.76 nm	Previous-publication parameter set
Membrane edge tension	*γ*	${1.8\times 10^{-11}}\hbox {J}\,\hbox {m}^{-1}$	^3^
Steric repulsion coefficient	*β*	${1.4\times 10^{-19}}\,\text {J}$	Previous-publication parameter set
Nuclear radius and envelope thickness	$r_{\textrm{nuc}}$, $d_{\textrm{nm}}$	${2}\,\upmu \hbox {m}$, 50 nm	Reference values; not fitted in single-shell model

### Microfluidic experimental and impedance-spectroscopy framework

#### Platform architecture and electrode configuration

**Fig. 3:**
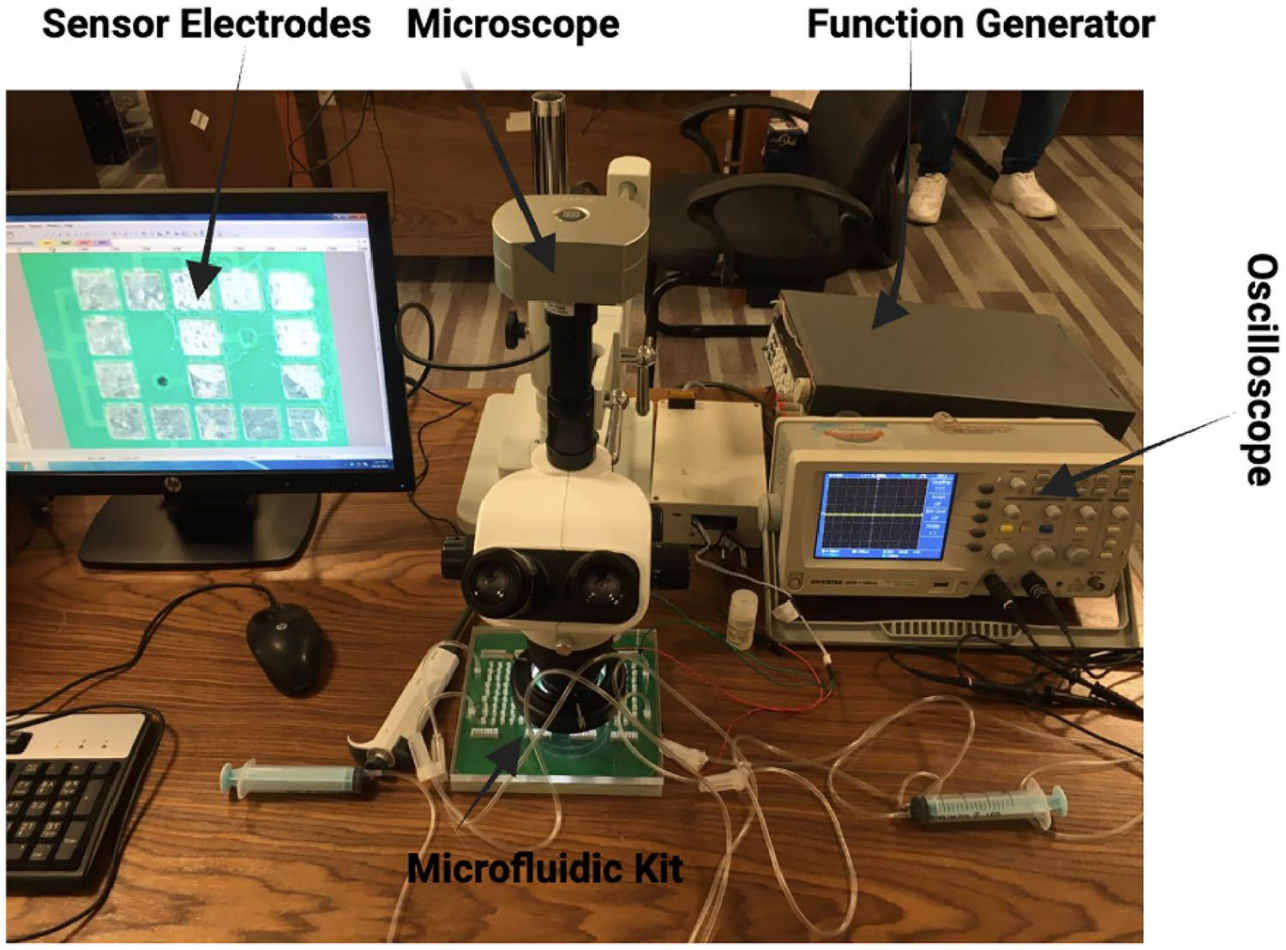
Laboratory photograph of the experimental microfluidic electroporation and impedance spectroscopy test bench. The setup includes a binocular optical microscope, a custom PDMS microchannel mounted on a routed PCB, a programmable nanosecond function generator, a four-channel digital oscilloscope, and a desktop computer for real-time visualization and waveform storage.

The proposed experimental platform for *in situ* extraction of electroporation parameters from stem cells is depicted in Fig. 3. The system integrates five core subsystems: (i)  Optical microscope positioned directly above the microfluidic chip for real-time optical monitoring of cell location; (ii) a custom-designed microfluidic kit comprising a polydimethylsiloxane (PDMS) microchannel bonded to a substrate that carries the coplanar electrode array, the assembly being mounted on a printed circuit board (PCB) for electrical routing; (iii) a function generator capable of producing step voltage pulses with sub-nanosecond rise times and peak amplitudes sufficient to establish the required electric field across the fabricated inter-electrode gap; (iv) a four-channel digital oscilloscope for time-resolved recording of voltage and current transients at the sensing electrodes; and (v) a desktop computer that displays the microscope video feed and stores the acquired waveform data for subsequent off-line feature extraction.

**Fig. 4:**
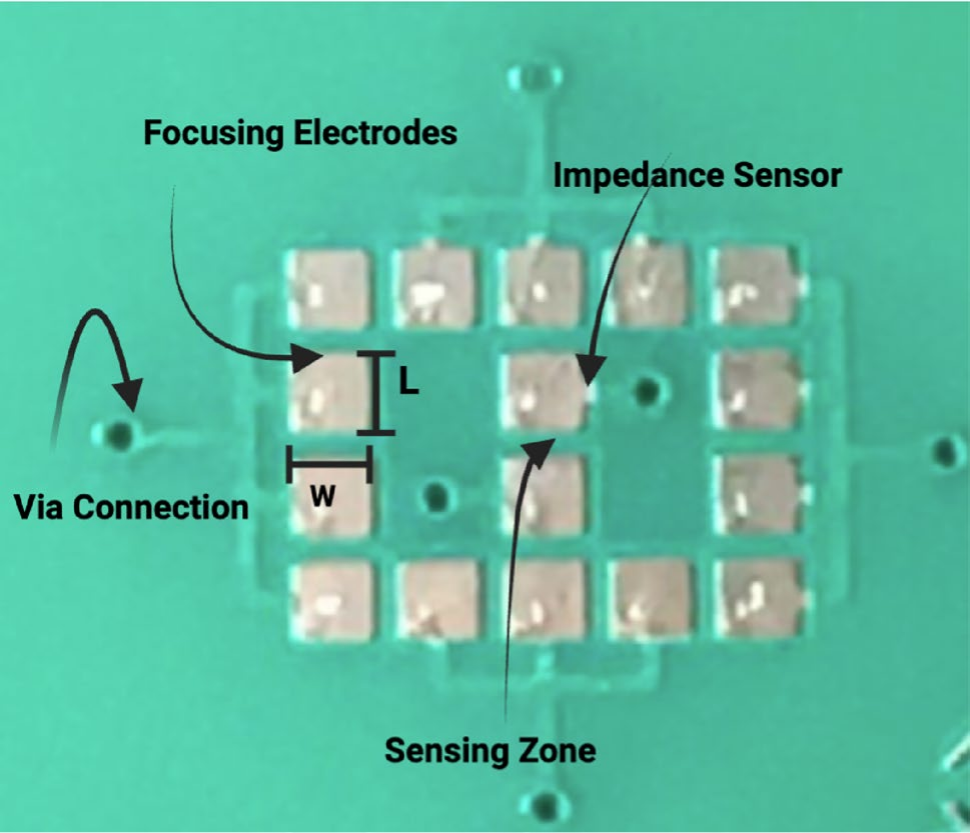
Optical micrograph of the coplanar sensing electrode array integrated into the microfluidic chip. Outer electrode pairs generate a non-uniform field for dielectrophoretic (DEP) trapping, and the central impedance-sensing pair consists of square electrodes with $W \times L = {600}\,\upmu \hbox {m} \times {600}\,\upmu \hbox {m}$ separated by a fabricated gap of $d = {200}\,\upmu \hbox {m}$.

The stem-cell suspension, prepared in phosphate buffered saline (PBS, $\sigma = {1}\,\hbox {S m}^{-1}$, $\varepsilon _r = 80$), is introduced into the PDMS microchannel via a precision syringe pump connected through polyethylene tubing. (see Fig. 4) trap and position a single stem cell between the impedance-sensor electrode pair. Once the cell is stably held in position confirmed by microscope observation on the computer monitor the excitation pulse is delivered from the function generator to the source electrode, while the ground electrode is held at zero potential. The resulting transient voltage and current waveforms are captured by the oscilloscope and transferred to the computer for extraction of the dielectric features.

Figure 4 presents an optical micrograph of the coplanar electrode array at higher magnification. The array is organized as a *4 × 4* grid of square metallic pads, with specific electrode pairs assigned to distinct functional roles. The *focusing electrodes*, enabling controlled positioning within the microchannel^21,22^. Because of fabrication constraints, the experimentally fabricated impedance-sensing electrodes had dimensions $W \times L = {600}\,\upmu \hbox {m} \times {600}\,\upmu \hbox {m}$ with an inter-electrode gap $d = {200}\,\upmu \hbox {m}$. The numerical model used a reduced representative gap of ${100}\,\upmu \hbox {m}$ to resolve the local electroporation field around the cell. Therefore, voltage-to-field conversions reported for the experimental impedance spectra are based on the fabricated ${200}\,\upmu \hbox {m}$ gap, whereas the COMSOL electroporation simulations are reported directly in terms of the applied electric field.

The *sensing zone*:the fluid-filled region between the two impedance-sensor electrodes is where the stem cell resides during the experiment; its volume is sufficiently small that the presence of a single cell measurably perturbs the inter-electrode impedance, providing the physical basis for single-cell electrical characterization. Metallized *via connections* route the electrode signals through the glass substrate to the PCB, which in turn interfaces with the function generator and oscilloscope via coaxial cables to minimize electromagnetic interference.

#### Cell preparation

Calibrated polystyrene microspheres (SPHERO, Inc.) with diameters of $10.4 {\pm } 0.2~\upmu \textrm{m}$ and $24.9 {\pm } 0.3~\upmu \textrm{m}$ were used as reference particles to verify the impedance sensor response before electroporation experiments. These two bead diameters were selected because they bracket the characteristic size range represented by the modelled hMSC geometries and provide a cell-free calibration for size-dependent impedance contrast in the same sensing geometry. The particles were suspended in deionized water ($\varepsilon _r = 78.5$, $\sigma _m = 5.5 \times 10^{-6}~\mathrm {S\,m^{-1}}$), and their known dielectric properties ($\varepsilon _p = 2.5$, $\sigma _p = 2.1 \times 10^{-4}~\mathrm {S\,m^{-1}}$) provided a controlled standard for reproducing impedance perturbations caused by particle size and position without the confounding effects of active membrane permeabilization. For each bead size, swept-frequency impedance was recorded in the same electrode configuration used for the stem cells. This calibration confirmed that the platform could resolve reproducible size-dependent impedance contrast before electroporation, thereby providing a traceable baseline for interpreting the subsequent voltage-dependent impedance drop caused by membrane permeabilization in stem cells.

#### Electrical excitation protocol

The excitation signal applied to the source electrode is a step function with a rise time of 1 ns. In the fabricated experimental geometry, the applied voltages of 1, 5, 10, 15, 20, and ${25}\,\text {V}$ correspond approximately to 50, 250, 500, 750, 1000, and ${1250}\,\hbox {V cm}^{-1}$, respectively, across the ${200}\,\upmu \hbox {m}$ gap. The numerical simulations use a prescribed field sweep of ${1}\,\hbox {kV cm}^{-1}$ to ${5}\,\hbox {kV cm}^{-1}$; therefore, the simulation range intentionally extends above the maximum experimental average field and should be interpreted as a mechanistic parameter sweep rather than a one-to-one matched-field validation. The nanosecond rise time charges the capacitive membrane rapidly relative to the pore formation time scale and is expected to limit bulk Joule heating in the conductive PBS medium under the selected conditions^10,24^. The pulse duration of ${1}\,\upmu \hbox {s}$ was selected to produce model-predicted permeabilization and to observe simulated post-pulse membrane recovery; direct viability and recovery assays were not performed in the present study.

#### Impedance spectroscopy and calibration

The single-shell RC model employed in this study has a direct and well-established connection to frequency-domain impedance spectroscopy^14–17,19,20^. Impedance spectroscopy was performed in the swept-frequency range of ${1}\,\hbox {kHz}$ to ${1}\,\hbox {MHz}$ using a precision impedance analyzer coupled to the microfluidic channel.

To isolate the true membrane impedance from system parasitics and electrode interface effects:

Calibration: Before cell trapping, the channel was filled with standard PBS solutions of known conductivity (${1.0}\,\hbox {S m}^{-1}$) to measure the open-channel baseline impedance. This calibration defines the geometry-dependent open-channel solution resistance. High-frequency residual values reported later are treated as frequency-dependent effective limits of the electrode–medium–cell system and are not used interchangeably with this baseline calibration resistance. The impedance analyzer was calibrated 10.4 *μ*m and 24.9 *μ*m polystyrene microbeads were measured as cell-free size standards.

**Table 4:** Microbead calibration used to verify the impedance-sensing workflow before hMSC measurements. Calibrated 10.4 *μ*m and 24.9 *μ*m polystyrene beads were used as cell-free size standards. The impedance magnitude was calculated using the 1 M*Ω* reference as $Z = 10^{6}\,\Omega \times (A_1/A_0)_{\textrm{measurement}} / (A_1/A_0)_{\textrm{reference}}$.

Microbead diameter	10 kHz	100 kHz	1 MHz
	M*Ω*	M*Ω*	M*Ω*
10.4 *μ*m	0.99997	0.99655	0.97801
24.9 *μ*m	1.00660	1.00624	1.00478

In the frequency domain, the cell membrane behaves as a capacitance-dominated element at low frequencies (where the membrane blocks ionic current, yielding high impedance magnitude |*Z*| and predominantly capacitive reactance $X_s$) and transitions to a conduction-dominated regime at higher frequencies (where displacement current shunts membrane resistance, yielding lower |*Z*| and predominantly resistive impedance $R_s$). During electroporation, the formation of hydrophilic pores increases the membrane conductance $G_m = \sigma _m/d_m$, introducing a parallel resistive pathway that shunts the membrane capacitance. This transition is directly related to the simulated dynamic membrane conductivity $\sigma _m(t)$: a cell undergoing electroporation is expected to exhibit a characteristic decrease in low-frequency |*Z*| and a shift of the crossover frequency to higher values, reflecting the increased membrane permeability. Thus, the time-domain conductivity features can serve as candidate predictive indicators for impedance-based detection of electroporation in microfluidic experiments^20,21^.

## Results and discussion

The simulation results are organized according to the four principal electroporation observables transmembrane potential, pore density, pore radius, and membrane conductivity examined for the undifferentiated hMSC model ($R_{\textrm{U}}={10}\,\upmu \hbox {m}$) and the osteogenic hMSC model ($R_{\textrm{O}}={13}\,\upmu \hbox {m}$). Membrane resealing dynamics are discussed separately.

### Transmembrane potential distribution

#### Undifferentiated model ($R_{\textrm{U}} = {10}\,\upmu \hbox {m}$)

**Fig. 5:**
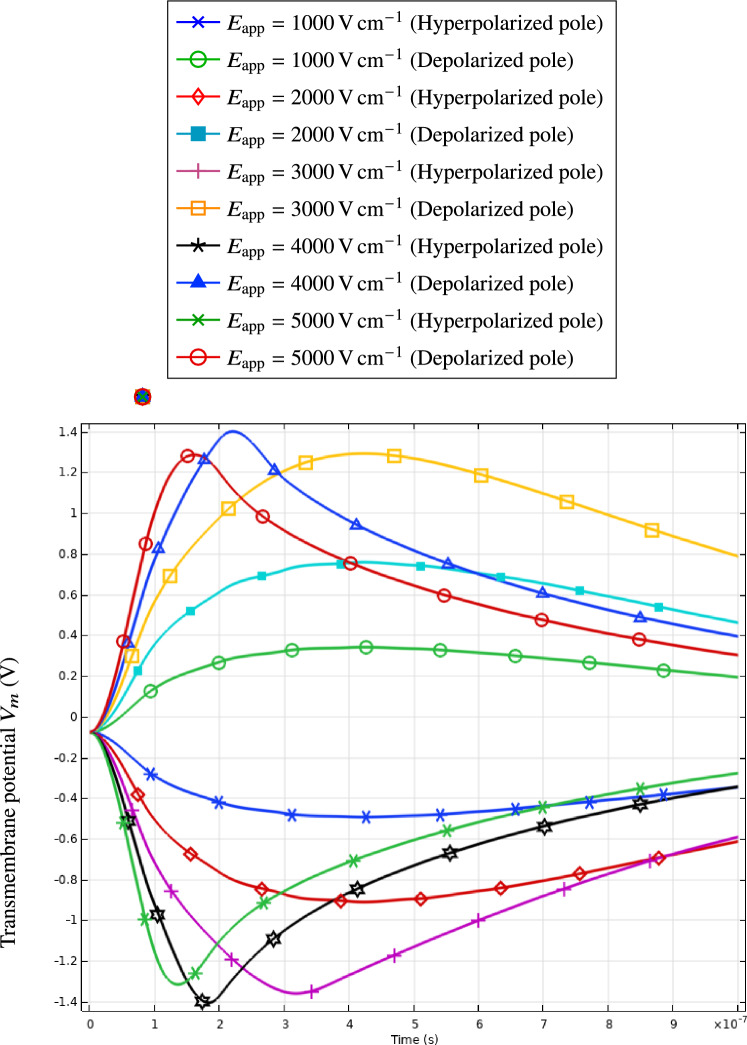
Transient transmembrane potential $V_m(t)$ of the undifferentiated hMSC model following application of a 5 kV/cm electroporation pulse. The hyperpolarized pole (H) develops the most negative transmembrane potential, whereas the depolarized pole (D) reaches the highest positive transmembrane potential. Intermediate membrane potentials are observed at the equatorial (border) regions, consistent with the angular dependence of membrane polarization predicted by Schwan’s equation. Using the electrical parameters, the passive Schwan RC time constant is estimated as $\tau _{\textrm{Schwan},U}\approx 4.69\times 10^{-7}$ s, while the theoretical steady-state Schwan driving voltage is approximately 7.5 V in the absence of electroporation. After the membrane reaches the electroporation threshold, the simulated response departs from passive RC charging because pore formation increases membrane conductance, thereby limiting further transmembrane potential buildup. The plotted post-threshold polar peaks are approximately ${\pm } 1.4$ V and are reached on a shorter, approximately ${1\times 10^{-7}}\,\text {s}$ to ${2\times 10^{-7}}\,\text {s}$ time scale. Compared with the osteogenic response in Fig. 6, the undifferentiated model has a lower passive Schwan driving voltage (${7.5}\,\text {V}$ vs. ${9.75}\,\text {V}$) and a shorter passive RC time constant, but the post-threshold peak amplitude is similar because electroporation clamps the membrane voltage.

Figure 5 presents the time-dependent transmembrane potential $V_m(t)$ recorded at all membrane segments of the undifferentiated hMSC model ($R_{\textrm{U}} = {10}\,\upmu \hbox {m}$). Upon application of the pulse, the membrane undergoes rapid capacitive charging. Using the previous-publication values $C_{m,\textrm{U}}=1\times 10^{-2}\,\hbox {F m}^{-2}$, $\sigma _{i,\textrm{U}}={0.32}\,\hbox {S m}^{-1}$, and $\sigma _e={0.32}\,\hbox {S m}^{-1}$, the passive Schwan RC estimate is $\tau _{\textrm{Schwan,U}}\approx {4.69\times 10^{-7}}\,\text {s}$. This analytical value is an intact-membrane reference, not the apparent rise time of the electroporating trace. In Fig. 5, the post-threshold polar peaks are reached on the order of ${1\times 10^{-7}}\,\text {s}$ to ${2\times 10^{-7}}\,\text {s}$. The passive polar transmembrane potential at ${5}\,\hbox {kV cm}^{-1}$ is approximately ${7.5}\,\text {V}$, far above the characteristic electroporation voltage $U_{\textrm{ep,U}}={0.258}\,\text {V}$. Therefore, the voltage trace should be interpreted as a rapid transition from passive charging to pore-mediated conductance rather than as a purely passive RC response.

#### Osteogenic model ($R_{\textrm{O}} = {13}\,\upmu \hbox {m}$)

**Fig. 6:**
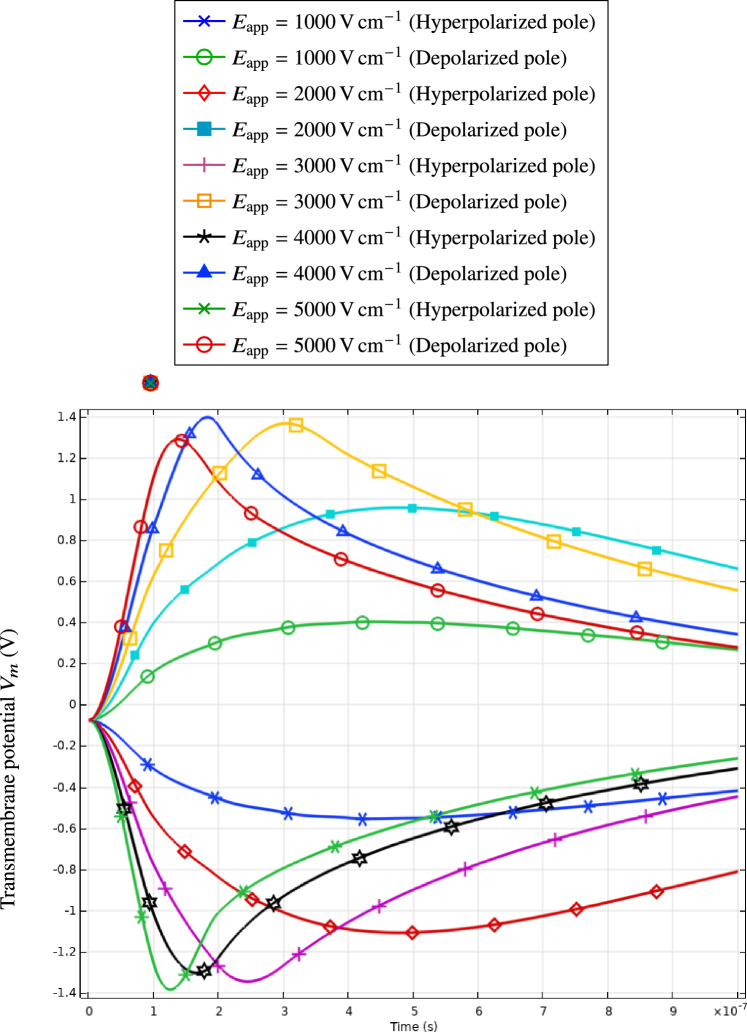
Transient transmembrane potential $V_m(t)$ of the osteogenic hMSC model subjected to a 5 kV/cm electroporation pulse. The simulated membrane potential rapidly rises to the electroporation threshold and subsequently becomes limited by pore formation, producing a peak value of approximately ${\pm }1.4$ V. For the corresponding intact-membrane Schwan model, the passive RC time constant is estimated as $\tau _{\textrm{Schwan},O}\approx 5.96\times 10^{-7}$ s, while the theoretical steady-state Schwan driving voltage is approximately 9.75 V. The discrepancy between the passive Schwan prediction and the simulated response reflects nonlinear membrane electroporation, where the formation of conductive pores limits further membrane charging beyond the electroporation threshold. The visible post-threshold rise in the plotted trace occurs on a shorter, approximately ${1\times 10^{-7}}\,\text {s}$ to ${2\times 10^{-7}}\,\text {s}$ time scale. Relative to the undifferentiated model in Fig. 5, the osteogenic model has the larger passive Schwan driving voltage (${9.75}\,\text {V}$ vs. ${7.5}\,\text {V}$) and the longer passive RC time constant, but both models converge to a similar clamped post-threshold voltage of approximately ${\pm }1.4$ V after pore formation begins.

Figure 6 presents the analogous transmembrane-potential waveforms for the osteogenic hMSC model ($R_{\textrm{O}} = {13}\,\upmu \hbox {m}$). With $C_{m,\textrm{O}}=8\times 10^{-3}\,\hbox {F m}^{-2}$ and $\sigma _{i,\textrm{O}}={0.24}\,\hbox {S m}^{-1}$, the passive Schwan RC estimate is $\tau _{\textrm{Schwan,O}}\approx {5.96\times 10^{-7}}\,\text {s}$, approximately 27% longer than the undifferentiated passive estimate. This analytical value should not be read as the apparent rise time in Fig. 6; the plotted electroporating traces reach their post-threshold peaks earlier, again on the order of ${1\times 10^{-7}}\,\text {s}$ to ${2\times 10^{-7}}\,\text {s}$. The passive polar voltage at ${5}\,\hbox {kV cm}^{-1}$ is approximately ${9.75}\,\text {V}$; however, pore-mediated conductance limits the physically sustained membrane potential after electroporation begins. The larger osteogenic radius increases the passive driving voltage, while its lower cytoplasmic conductivity and different membrane capacitance slow the intact-membrane RC response.

### Pore density evolution

#### Undifferentiated model ($R_{\textrm{U}} = {10}\,\upmu \hbox {m}$)

**Fig. 7:**
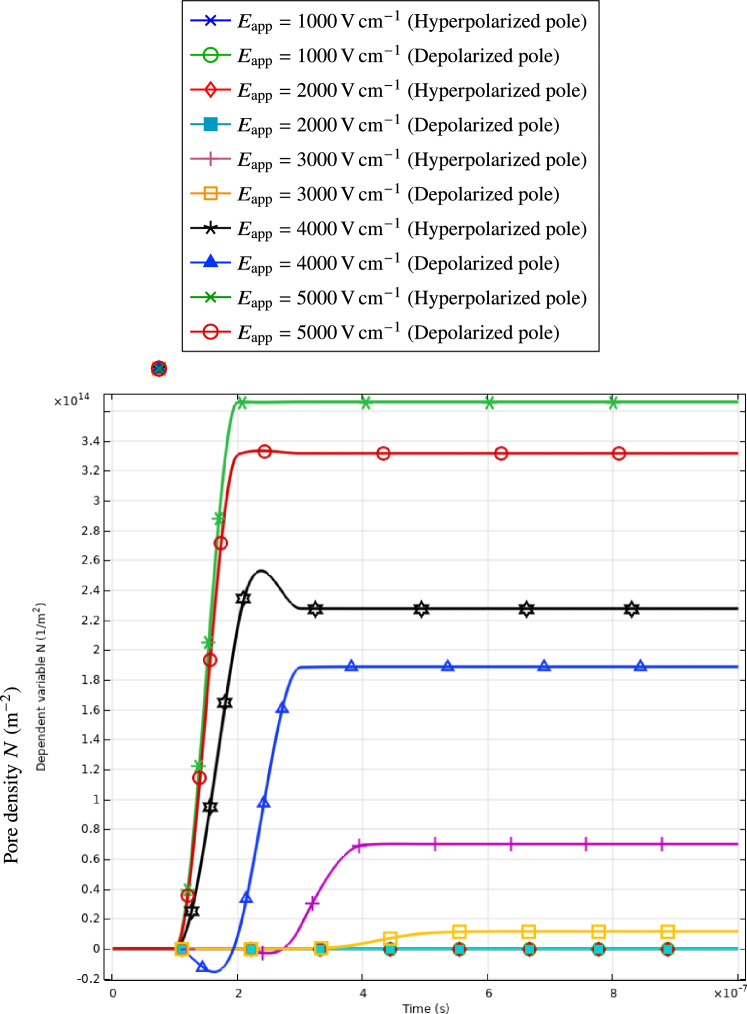
Pore density *N*(*t*) at different membrane positions of the undifferentiated hMSC model following a ${5}\,\hbox {kV cm}^{-1}$ electroporation pulse. The hyperpolarized and depolarized poles reach comparable maximum pore densities that are higher than the border regions, consistent with the angular dependence of $V_m$ and the exponential dependence of pore creation on $(V_{\textrm{TM}}/U_{\textrm{ep}})^2$. Compared with the osteogenic case in Fig. 8, the undifferentiated model shows a slightly higher maximum polar pore-density trace (approximately 3.6–$3.7\times 10^{14}\ \textrm{m}^{-2}$) but lower pore-density levels in several non-polar/border traces. This indicates that the cell-type difference is not only the polar maximum, but also how pore formation spreads across membrane segments.

Figure 7 illustrates the time-dependent distribution of pore density *N*(*t*) across the membrane segments of the undifferentiated hMSC model. The highest pore density occurs at the polar membrane regions, in agreement with the established theoretical framework for electroporation: because the poles experience the largest transmembrane-potential magnitude, the exponential dependence of the pore nucleation rate on $(V_{\textrm{TM}}/U_{\textrm{ep}})^2$ in Eq. (4) yields the most rapid pore formation at these locations. The undifferentiated threshold field estimated from $U_{\textrm{ep,U}}/(1.5R_{\textrm{U}})$ is approximately ${0.172}\,\hbox {kV cm}^{-1}$, so all simulated fields from ${1}\,\hbox {kV cm}^{-1}$ to ${5}\,\hbox {kV cm}^{-1}$ are supra-threshold.

#### Osteogenic model ($R_{\textrm{O}} = {13}\,\upmu \hbox {m}$)

**Fig. 8:**
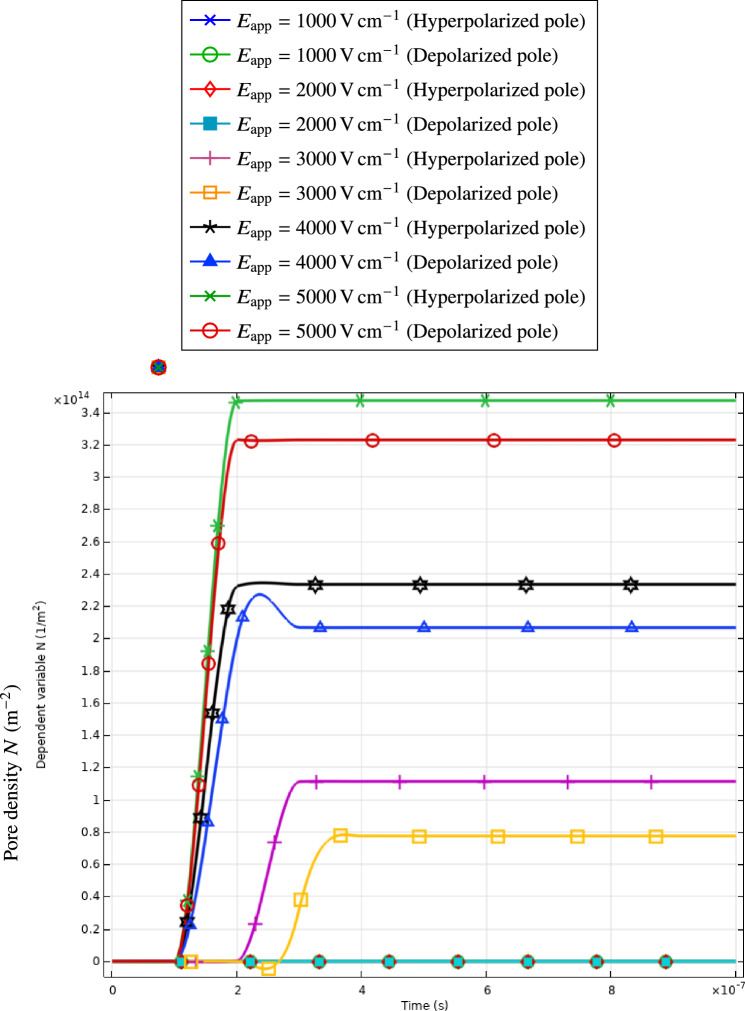
Pore density *N*(*t*) at different membrane positions of the osteogenic hMSC model following a ${5}\,\hbox {kV cm}^{-1}$ electroporation pulse. The polar membrane regions show the strongest pore-density response, whereas border regions remain lower because the local transmembrane potential is smaller. This spatial trend supports the use of pole-specific pore-density descriptors when comparing the two cell types. Relative to the undifferentiated model in Fig. 7, the osteogenic response reaches a similar but slightly lower polar maximum (about 3.4–$3.5\times 10^{14}\ \textrm{m}^{-2}$) and shows more elevated intermediate/border pore-density plateaus in some traces. Thus, the osteogenic profile is distinguished more by the distribution and timing of pore creation across membrane segments than by a large increase in the absolute polar maximum.

The pore density evolution for the osteogenic hMSC model is presented in Fig. 8. The time-dependent pore distribution analysis indicates that the highest values again occur at the polar membrane regions, in agreement with the established electroporation theory. The osteogenic threshold field estimated from $U_{\textrm{ep,O}}/(1.5R_{\textrm{O}})$ is approximately ${0.164}\,\hbox {kV cm}^{-1}$, slightly lower than the undifferentiated threshold because the larger radius offsets the higher characteristic electroporation voltage. The osteogenic pore-density response is therefore expected to be strongly activated throughout the simulated field range, but the lower cytoplasmic conductivity and altered capacitance modify the charging trajectory before pore creation becomes dominant.

### Pore radius characterization

#### Undifferentiated model ($R_{\textrm{U}} = {10}\,\upmu \hbox {m}$)

**Fig. 9:**
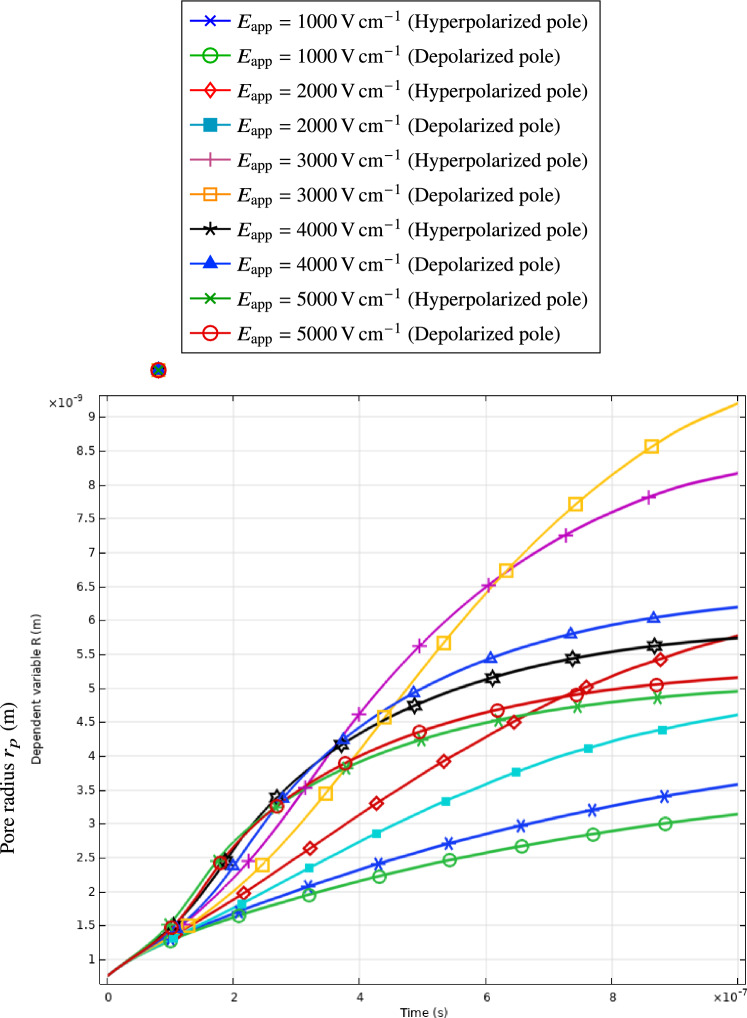
Pore radius $r_p(t)$ at different membrane positions of the undifferentiated hMSC model for applied electric-field strengths from ${1}\,\hbox {kV cm}^{-1}$ to ${5}\,\hbox {kV cm}^{-1}$. Within the plotted early-time window, the visible pore radii reach approximately ${9.2}\,\text {nm}$ at the largest trace and span roughly ${3.1}\,\text {nm}$ to ${9.2}\,\text {nm}$ near the right edge of the plot. The traces show position- and field-dependent pore expansion and should not be described as strictly monotonic with applied field in this exported figure.

The formation of hydrophilic pores on the cell membrane surface is the principal manifestation of the membrane’s response to electroporation in stem cells. These pores enable the membrane to act as a conduit for delivering ions, DNA, and other large biomolecules into the cell, making pore radius a critical parameter for optimizing electroporation-mediated delivery protocols. Fig. 9 presents the time-dependent pore radius $r_p(t)$ at different membrane positions for the undifferentiated hMSC model across the range of applied electric field strengths (${1}\,\hbox {kV cm}^{-1}$ to ${5}\,\hbox {kV cm}^{-1}$).

In the plotted early-time window, the pore-radius traces span approximately ${3.1}\,\hbox {nm}$ to ${9.2}\,\hbox {nm}$ by the right edge of Fig. 9, with the largest visible trace reaching $r_p\approx {9.2}\,\hbox {nm}$. Because the displayed curves are still increasing near the right edge of the early-time window, this value is not interpreted as a true global maximum over the full ${1}\,\upmu \hbox {s}$ pulse. It represents the largest pore-radius value extracted from the early expansion interval shown in Fig. 9. The exported traces should be interpreted as position- and field-dependent simulation outputs rather than as a strictly monotonic ordering with applied field.

#### Osteogenic Model ($R_{\textrm{O}} = {13}\,\upmu \hbox {m}$)

**Fig. 10:**
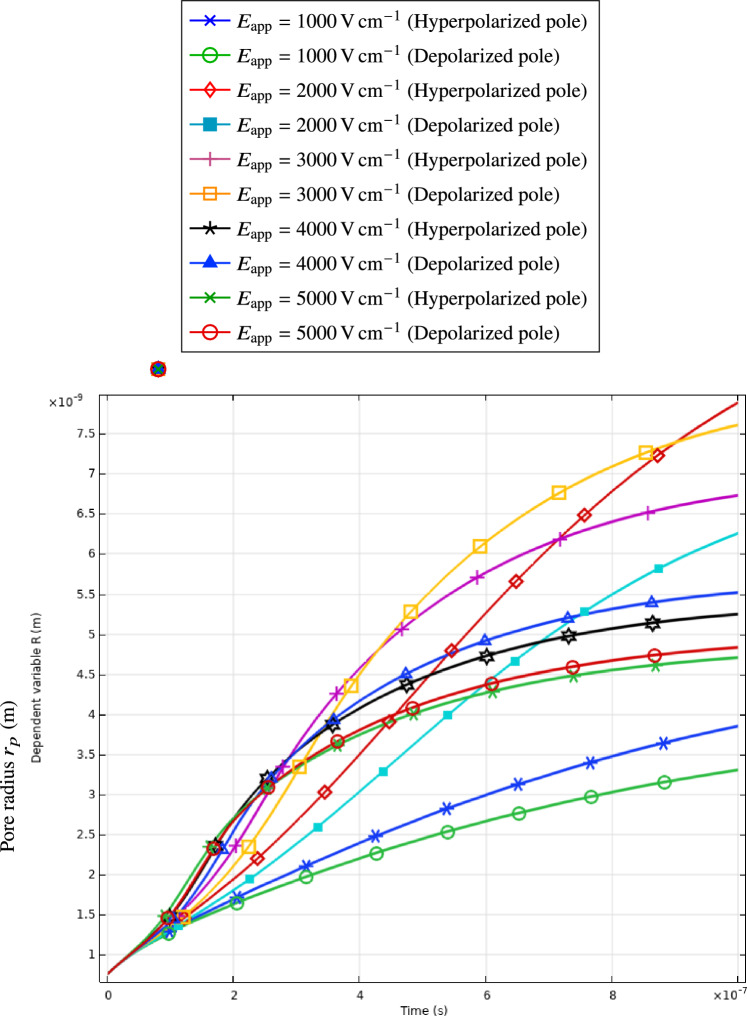
Pore radius $r_p(t)$ at different membrane positions of the osteogenic hMSC model for applied electric-field strengths from ${1}\,\hbox {kV cm}^{-1}$ to ${5}\,\hbox {kV cm}^{-1}$. Within the plotted early-time window, the visible pore radii reach approximately ${7.9}\,\text {nm}$ at the largest trace and span roughly 3.3 nm to 7.9 nm near the right edge of the plot. The traces indicate electroporation-mediated pore expansion across membrane positions, but their exported ordering is not strictly monotonic with applied field.

Figure 10 presents the pore radius evolution for the osteogenic hMSC model. In the plotted early-time window, the visible pore-radius traces span approximately 3.3 nm to 7.9 nm near the right edge of the plot, with the largest visible trace reaching $r_p\approx {7.9}\,\text {nm}$. The main supported observation is time-dependent pore expansion across membrane positions; the exported trace ordering should not be interpreted as a strictly monotonic field-strength dependence.

### Membrane conductivity dynamics

#### Undifferentiated model ($R_{\textrm{U}} = {10}\,\upmu \hbox {m}$)

**Fig. 11:**
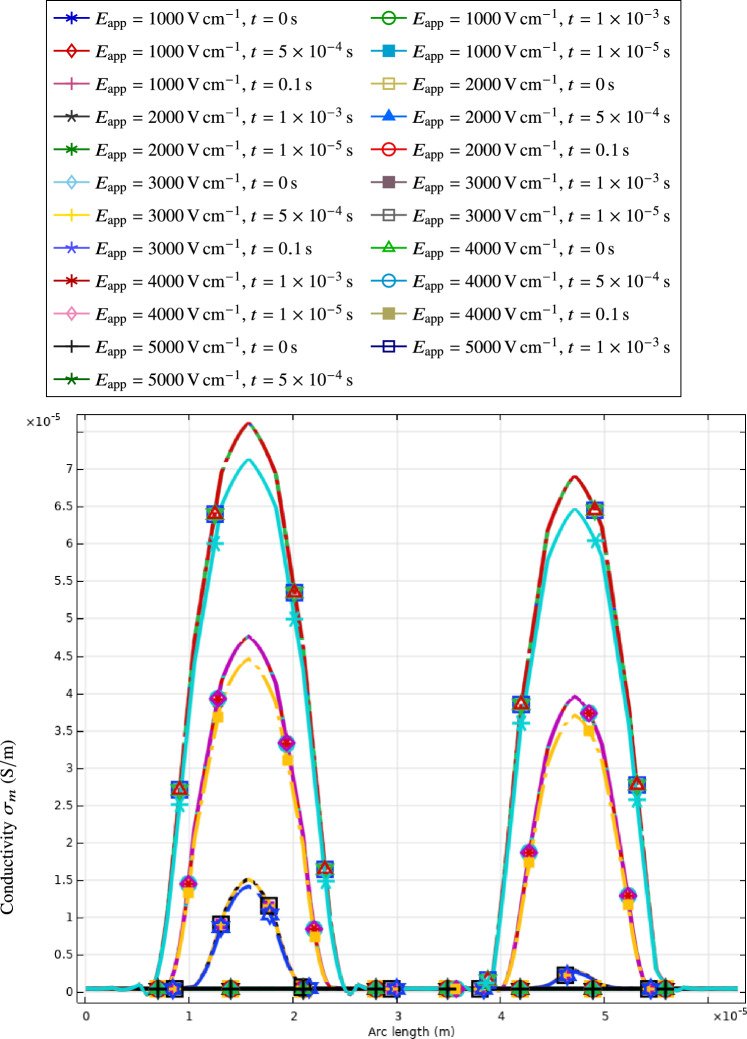
Spatial distribution of membrane conductivity $\sigma _m$ along the membrane perimeter of the undifferentiated hMSC model at selected time points. The x-axis represents boundary arc length in meters, with pole positions determined by the chosen arc-length origin. The conductivity peaks identify the polar membrane regions facing the excitation electrodes, where pore-mediated conductance is largest.

**Fig. 12:**
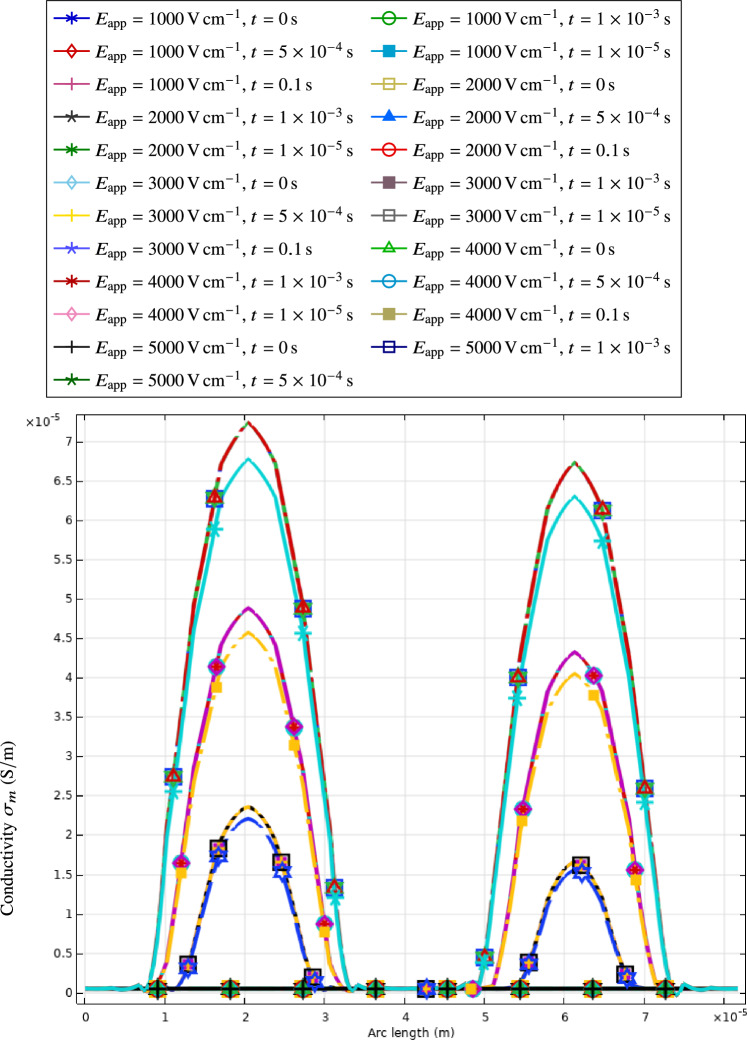
Spatial distribution of membrane conductivity $\sigma _m$ along the membrane perimeter of the osteogenic hMSC model at selected time points. The x-axis represents boundary arc length in meters, with pole positions determined by the chosen arc-length origin. The two dominant conductivity maxima correspond to the polar regions facing the excitation electrodes and indicate localized electroporation-induced membrane conductance.

When stem cell membranes are exposed to an electric field of adequate strength, local electrical conductivity increases as hydrophilic pores form and expand. Figs.  11 and 12 present spatial distributions of $\sigma _m$ along the membrane perimeter at selected field strengths and sampled times. The figs should be interpreted as spatial profiles at discrete simulation states, not continuous time traces. The x-axis in the plotted images is arc length in meters along the membrane boundary; the corresponding full perimeter is approximately *2π R*. Pole locations depend on the chosen arc-length origin and are therefore described qualitatively rather than by normalized positions in these figures. The peak conductivities are on the order of $10^{-4}\ \mathrm {S/m}$ for the selected high-field profiles. Because the displayed conductivity figs include selected profiles only up to ${3}\,\hbox {kV cm}^{-1}$, values extracted from separate ${5}\,\hbox {kV cm}^{-1}$ simulations are identified in tables as model outputs rather than read directly from these plotted profiles.

#### Osteogenic model ($R_{\textrm{O}} = {13}\,\upmu \hbox {m}$)

Figure 12 presents the corresponding spatial conductivity profiles along the membrane perimeter for the osteogenic hMSC model. The two-maxima profile is physically consistent with polar electroporation regions generated by the non-uniform electric field. As for the undifferentiated hMSC model, the Fig. displays selected spatial profiles rather than a complete time history; peak values reported elsewhere for ${5}\,\hbox {kV cm}^{-1}$ should therefore be interpreted as extracted simulation outputs rather than direct readings from this particular Fig.

### Membrane resealing behavior

The electroporation process for stem cells can be categorized into three primary states: the non-permeabilized state, the permeabilized state, and the recovery (resealing) state. The non-permeabilized state describes the membrane condition before application of any external electric field, where the equilibrium pore density is $N_0$ and the membrane conductivity is at its baseline value. Upon exposure to an electric field of sufficient strength, the permeabilized state is initiated when the membrane potential exceeds the threshold voltage ($V_{\textrm{th}} \approx {0.2}\,\text {V}$ to ${1}\,\text {V}$), resulting in a dramatic increase in pore density and transmembrane potential. For the stem cells investigated here, reaching the permeabilized state requires an electric field in the ${1\times 10^{3}}\,\hbox {V cm}^{-1}$ to ${1\times 10^{4}}\,\hbox {V cm}^{-1}$ range, with the exact threshold depending on the specific cell dimensions. Following the cessation of the electric pulse, the recovery state begins as the induced potential falls below the irreversible breakdown level and the membrane gradually reseals.

#### Resealing dynamics of the undifferentiated model

**Fig. 13:**
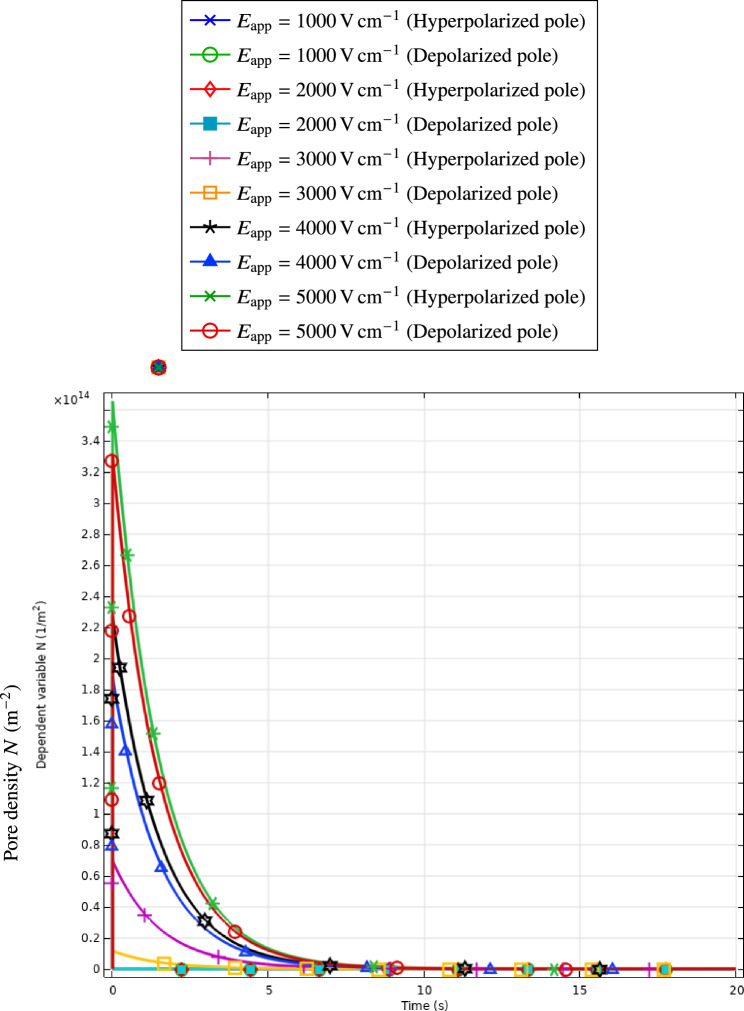
Membrane resealing dynamics of the undifferentiated hMSC model following exposure to a ${5}\,\hbox {kV cm}^{-1}$ electroporation pulse. After pulse cessation, the transmembrane potential, pore density, and membrane conductivity decay progressively toward their pre-stimulation values, indicating model-predicted post-pulse membrane recovery under the selected pulse parameters. Compared with the osteogenic recovery in Fig. 14, the undifferentiated case should be read as the reference recovery curve: the decay is gradual, leaving residual pore-density/conductivity during the displayed recovery interval rather than an immediate return to the baseline.

Figure 13 illustrates the membrane resealing dynamics of the undifferentiated hMSC model following exposure to the electric stimulus pulse. The time-decay curves demonstrate that the membrane potential, pore density, and conductivity all decrease progressively after pulse termination, approaching (but not immediately reaching) their pre-stimulation baseline values. This resealing behavior is critical for maintaining cellular integrity and avoiding permanent deformation or cell death. Under the selected electric pulse parameters, the model predicts reversible resealing, suggesting that the protocol may be compatible with viability-preserving delivery workflows. However, in the absence of direct biological viability assays (such as propidium iodide or calcein-AM staining) in the current study, these results remain model-based predictions of reversibility, and future experimental work is required to establish the cell survival rate under these conditions.

#### Resealing dynamics of the osteogenic model

**Fig. 14:**
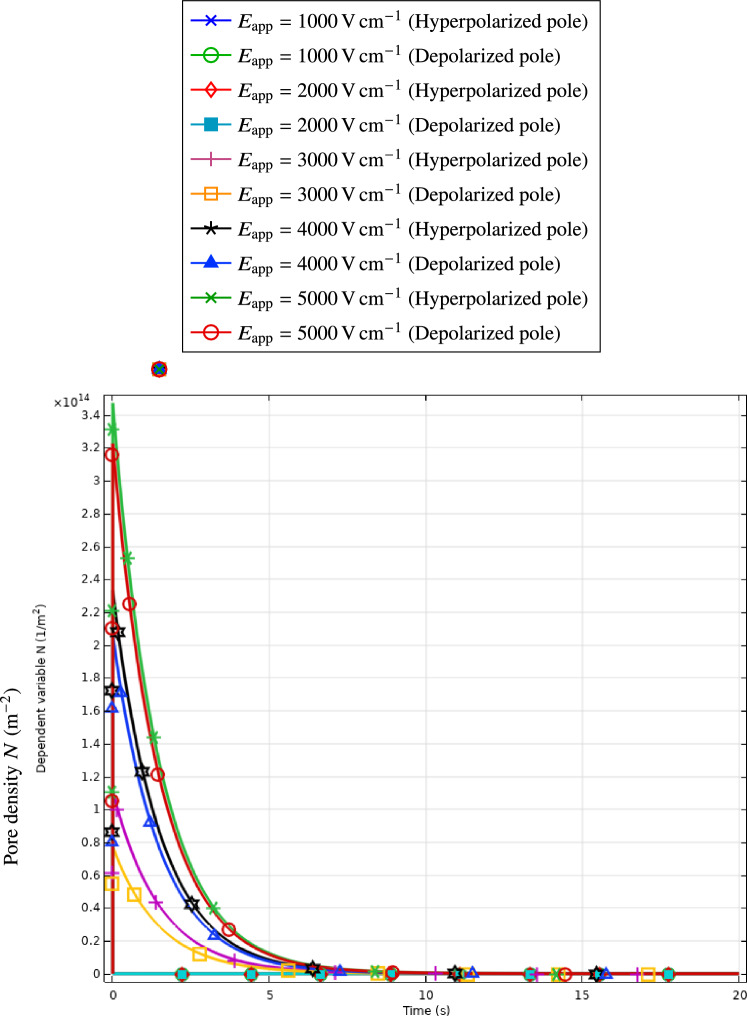
Membrane resealing dynamics of the osteogenic hMSC model following exposure to a ${5}\,\hbox {kV cm}^{-1}$ electroporation pulse. The post-pulse recovery of transmembrane potential, pore density, and membrane conductivity is consistent with model-predicted membrane resealing. Comparison with the undifferentiated hMSC response indicates cell-type-dependent recovery kinetics that may serve as additional candidate descriptors for hMSC characterization. Relative to Fig. 13, the osteogenic model shows a different recovery trajectory, with the initial post-pulse decline occurring more rapidly in the model interpretation while residual pore-density/conductivity differences remain during the plotted recovery window. The key contrast is therefore the resealing rate and residual recovery level, not merely the presence or absence of resealing.

Figure 14 presents the corresponding resealing dynamics for the osteogenic hMSC model. The qualitative resealing behavior is similar to the undifferentiated hMSC model; however, quantitative differences in the resealing time constant and the residual pore density after a given recovery period provide additional discriminative features between the two cell-type models. The osteogenic hMSC model exhibits a slightly more rapid initial phase of conductivity recovery, consistent with the higher peak conductivity (and hence stronger thermodynamic driving force for pore closure) observed during the pulse phase.

### Consolidated quantitative results

Table 5 consolidates the parameter-consistent numerical comparison between the two biological cell-type models. The values are calculated from the previous-publication parameters. These quantities define the passive charging and electroporation-threshold context for interpreting the pore-density, pore-radius, and conductivity plots. Because pore-mediated conductance strongly alters the membrane after threshold crossing, the passive Schwan voltage should be read as the pre-poration driving voltage, not as a sustained post-poration membrane voltage.

**Table 5:** Cell-type parameter and derived-model comparison for undifferentiated and osteogenic models. Passive polar $V_m$ is calculated at ${5}\,\hbox {kV cm}^{-1}$; threshold field is estimated as $U_{\textrm{ep}}/(1.5R)$.

Parameter	Undifferentiated	Osteogenic	Interpretation
Representative radius	${10}\,\upmu \hbox {m}$	${13}\,\upmu \hbox {m}$	Osteogenic model is 30% larger
Cytoplasmic conductivity $\sigma _i$	${0.32}\,\hbox {S m}^{-1}$	${0.24}\,\hbox {S m}^{-1}$	Osteogenic cytoplasm is 25% less conductive
Specific membrane capacitance $C_m$	$1\times 10^{-2}\,\hbox {F m}^{-2}$	$8\times 10^{-3}\,\hbox {F m}^{-2}$	Osteogenic value is 20% lower
Characteristic electroporation voltage $U_{\textrm{ep}}$	${0.258}\,\text {V}$	${0.32}\,\text {V}$	Osteogenic threshold voltage is 24.0% higher
Passive Schwan RC time $\tau _{\textrm{Schwan}}$	${4.69\times 10^{-7}}\,\text {s}$	${5.96\times 10^{-7}}\,\text {s}$	Intact-membrane analytical reference
Apparent plotted post-threshold rise time	On the order of $1\times 10^{-7}\,\text {s}$ to ${2\times 10^{-7}}\,\text {s}$		Read from Figs. 5, 6; shorter than passive RC estimate
Passive polar $V_m$ at ${5}\,\hbox {kV cm}^{-1}$	${7.5}\,\text {V}$	${9.75}\,\text {V}$	Osteogenic passive drive is 30% higher
Estimated threshold field	${0.172}\,\hbox {kV cm}^{-1}$	${0.164}\,\hbox {kV cm}^{-1}$	Larger radius slightly offsets higher $U_{\textrm{ep}}$
Initial membrane conductivity $\sigma _{m,0}$	${5\times 10^{-7}}\,\hbox {S m}^{-1}$		Derived from $G_m d_m$
Initial membrane resistance per area	${0.01}\,\Omega \,\hbox {m}^{2}$		Derived from $d_m/\sigma _{m,0}$

**Table 6:** Numerical comparison between the model parameters and the measured impedance response. Percentage change is calculated as $(\textrm{Osteogenic}-\textrm{Undifferentiated})/\textrm{Undifferentiated}\times 100\%$. Experimental impedance values are the mean values measured at ${1}\,\hbox {kHz}$.

Feature	Undifferentiated hMSC	Osteogenic hMSC	Difference	Change
Representative radius	${10}\,\upmu \hbox {m}$	${13}\,\upmu \hbox {m}$	+${3}\,\upmu \hbox {m}$	+30.0%
$\tau _{\textrm{Schwan}}$ from parameters	${4.69\times 10^{-7}}\,\text {s}$	${5.96\times 10^{-7}}\,\text {s}$	$+{1.27\times 10^{-7}}\,\text {s}$	+27.1%
Apparent plotted post-threshold rise time	$1\times 10^{-7}\,\text {s}\hbox { to }{2\times 10^{-7}}\,\text {s}$ scale		Shorter than passive Schwan estimate	
Passive polar $V_m$ at ${5}\,\hbox {kV cm}^{-1}$	${7.5}\,\text {V}$	${9.75}\,\text {V}$	+${2.25}\,\text {V}$	+30.0%
Estimated threshold field	${0.172}\,\hbox {kV cm}^{-1}$	${0.164}\,\hbox {kV cm}^{-1}$	$-{0.008}\,\hbox {kV cm}^{-1}$	*-4.6%*
|*Z*| at 1 V, $${1}\,\hbox {kHz}$$	$${7863}\,\Omega$$	$${11\,029}\,\Omega$$	$$+{3166}\,\Omega$$	+40.3%
|*Z*| at 25 V, $${1}\,\hbox {kHz}$$	$${599}\,\Omega$$	$${1448}\,\Omega$$	$$+{849}\,\Omega$$	+141.7%
Impedance drop at 25 V	92.4%	86.9%	*-5.5* pp	Lower in osteogenic cells
Crossover frequency at 25 V	${121.9}\,\hbox {kHz}$	${52.2}\,\hbox {kHz}$	$-{69.7}\,\hbox {kHz}$	*-57.2%*

### Quantitative model-data comparison and parameter sensitivity analysis

The membrane conductivity obtained from the electroporation model provides a physical explanation for the impedance variations observed experimentally. Before electroporation, the intact cell membrane exhibits very low conductivity and high electrical resistance, limiting current flow at low frequencies and producing the characteristic membrane polarization. As the applied electric field induces pore formation, the membrane conductivity increases, reducing the membrane resistance and allowing greater current conduction through the membrane. Consequently, the measured impedance magnitude decreases, particularly at low frequencies where the membrane contribution is most significant. This behavior provides a direct physical interpretation of the impedance spectra obtained after electroporation.

Upon application of a supra-threshold electric field, the electroporation equations predict a rapid rise in pore density and pore radius, which increases $\sigma _m(t)$ and reduces the effective membrane resistance. In the equivalent electrical circuit (Fig. 2), this reduction in $R_m$ shunts the parallel membrane capacitance $C_m$, allowing more low-frequency current to pass through or around the permeabilized cell. In the measured spectra, this same physical pathway appears as a reduction in low-frequency impedance magnitude |*Z*| by 92.4 % for undifferentiated hMSCs and 86.9 % for osteogenic hMSCs at ${25}\,\text {V}$ (Table 7). This comparison supports the proposed physical link between simulated conductivity dynamics and measured impedance drops, although it does not by itself constitute a full quantitative validation of every model state variable.

To assess model robustness, a parameter sensitivity analysis was performed by perturbing key biophysical and electrical inputs by ${\pm } 10\%$ or ${\pm } 20\%$. This analysis is interpreted in two regimes: the passive/pre-poration regime, where Schwan-type radius scaling is expected, and the post-threshold clamped regime, where pore-mediated conductance limits the polar voltage:

1. Membrane capacitance ($C_m$): the cell-type values give $C_{m,\textrm{U}}=1\times 10^{-2}\,\hbox {F m}^{-2}$ and $C_{m,\textrm{O}}=8\times 10^{-3}\,\hbox {F m}^{-2}$. Because $\tau _m$ is proportional to $C_m$, capacitance primarily dictates the transient charging rate before pore-mediated conductance dominates.
2. Cytoplasmic conductivity ($\sigma _c$): perturbing the cytoplasmic conductivity $\sigma _c$ by ${\pm } 20\%$ (from ${0.24}\,\hbox {S m}^{-1}$ to ${0.36}\,\hbox {S m}^{-1}$) alters the internal electric field shielding. This perturbation results in an inverse shift of ${\mp } 4.5\%$ in the charging time $\tau _{\textrm{charge}}$ and shifts the peak transmembrane potential reached during the transient charging phase by ${\pm } 1.2\%$.
3. Extracellular medium conductivity ($\sigma _e$): the parameter set uses $\sigma _e={0.32}\,\hbox {S m}^{-1}$ for PBS. Changes in this value alter current distribution, baseline solution resistance, and charging time, so PBS conductivity is treated as a critical calibration parameter rather than a free fitting term.
4. Membrane conductivity ($\sigma _{m,0}$ and $\sigma _m(t)$): the baseline value $\sigma _{m,0}={5\times 10^{-7}}\,\hbox {S m}^{-1}$ sets the intact-membrane resistance, while pore-mediated increases in $\sigma _m(t)$ dominate the post-threshold impedance response. The experimental collapse of low-frequency |*Z*|, $R_s$, and $|X_s|$ with applied voltage is therefore interpreted as the frequency-domain signature of increasing membrane conductance.
5. Cell radius (*R*): in the passive/pre-poration regime, radius remains a dominant determinant of the induced transmembrane potential because $V_m \propto R$ in Eq. (2). The osteogenic radius is 30% larger than the undifferentiated radius, giving a 30% higher passive polar voltage at the same applied field.

This sensitivity analysis indicates that transient parameters such as the charging time are moderately sensitive to capacitive and medium properties, while the post-threshold clamped voltage and conductivity descriptors vary less under the selected perturbations. These outputs should be interpreted as model descriptors whose experimental utility requires matched-field validation.

## Comparative analysis

The hybrid analytical–numerical framework developed in this study advances the state of the art in electroporation modeling in several respects. DeBruin and Krassowska^3^ presented one of the first comprehensive single-cell electroporation models, demonstrating the exponential dependence of pore density on transmembrane potential and establishing the importance of the rest potential. However, their analysis was confined to a single cell geometry and did not extract quantitative feature sets suitable for cell characterization. The present work extends this foundational framework by introducing pole-specific charging time and post-threshold clamped voltage as candidate effective descriptors and by systematically comparing undifferentiated and osteogenic hMSC models within a realistic microfluidic geometry.

Retelj et al.^5^ investigated the electroporation of intracellular liposomes using nanosecond pulses, demonstrating the ability of short-duration fields to permeabilize organelle membranes. Their theoretical analysis employed a similar pore-density evolution equation; however, the study focused on intracellular organelles rather than the plasma membrane of intact cells and did not address the extraction of quantitative electroporation features for cellular characterization. The present model complements their work by focusing on the plasma membrane response of intact stem cells and correlating pore dynamics with measurable electrical signatures.

Garcia et al.^7^ performed a numerical investigation of electric and thermal cell-kill distributions in electroporation-based therapies at the tissue scale. While their work provided valuable insights into therapeutic planning, the tissue-level modeling inherently sacrifices single-cell resolution. In contrast, the present study operates at the single-cell level within a microfluidic channel, enabling the extraction of cell-specific features that are inaccessible in tissue-scale models.

Hanna et al.^9^ quantitatively studied the electropermeabilization of inner and outer cell membranes with microsecond pulsed electric fields using calcium-ion flux as a reporter. Their experimental measurements validated the concept that pulse parameters can selectively permeabilize different membrane compartments. The present computational framework is consistent with their experimental findings and provides a complementary theoretical tool for predicting membrane permeabilization under nanosecond pulse conditions where direct calcium imaging may be technically challenging.

Previous work by the present authors on impedance analysis and dielectrophoretic characterization of leukemic cells^20–22^ demonstrated that impedance-derived features can discriminate between normal and malignant white blood cells. The present study extends this paradigm to stem cells by relating model-derived time-domain electroporation features to frequency-domain impedance spectra. The RC-circuit analogy established herein (“Impedance spectroscopy and calibration”) provides a direct physical bridge between the time-domain features ($\tau _{\textrm{charge}}$, $V_{\textrm{clamp}}$, $\sigma _{m,\max }$) and the frequency-domain impedance parameters (|*Z*|, $R_s$, $X_s$) that can be measured experimentally, thereby unifying the two characterization approaches within a single modeling framework.

The key advantages of the present approach relative to existing methods may be summarized as follows. First, the hybrid analytical–numerical formulation combines the physical transparency of the analytical transmembrane potential expression (Eq. (2)) with the spatiotemporal resolution of the finite-element solver. Second, the coupling of the pore-radius evolution kinetics (Eq. (5)) with the pore-density equation (Eq. (4)) enables physically consistent modelling of pore expansion and resealing, which is necessary for relating time-domain observables to frequency-domain impedance features. Third, the introduction of pole-specific charging time and clamped voltage as cell-type descriptors enriches the candidate feature space beyond conventional pore density and transmembrane potential. Fourth, the systematic analysis of membrane conductivity dynamics and resealing kinetics provides candidate indicators for impedance-based monitoring of electroporation in microfluidic platforms. Fifth, the algorithmic framework (Algorithm 1) codifies the simulation and feature-extraction pipeline, facilitating reproducibility and extension to other cell types.

Taken together, the time-domain simulation features and the frequency-domain impedance measurements form a complementary, multi-modal combined feature space

6 $$\begin{aligned} {\mathcal {F}}_{\textrm{combined}} = \bigl \{ \tau _{\textrm{charge}},\; V_{\textrm{clamp}},\; N_{\max },\; r_{p,\max },\; \sigma _{m,\max },\; f_c,\; \Delta |Z|_{f_{c,0}},\; R_{s,1\,\textrm{kHz}},\; |X_{s,\textrm{pk}}| \bigr \}, \end{aligned}$$

which unifies mechanistic electroporation observables with experimentally measurable impedance biomarkers. The nine features in ${\mathcal {F}}_{\textrm{combined}}$ are defined in “Results and discussion” and “Impedance-based experimental characterization of stem cells under electroporation” and compared with the impedance measurements in “Consolidated impedance feature comparison”. This combined feature space is proposed as a multi-dimensional descriptor for future machine-learning studies, as it captures both intrinsic cell properties (through sub-threshold impedance features) and electroporation-induced changes (through supra-threshold features and time-domain observables).

## Impedance-based experimental characterization of stem cells under electroporation

To bridge the time-domain electroporation simulations presented in “Results and discussion” with measurable electrical signatures, swept-frequency impedance spectroscopy was performed on human mesenchymal stem cells (hMSCs) using the microfluidic platform described in “Methods” (Figs. 3 and 4). These two populations serve as experimental surrogates for the two cell-type models studied computationally: differentiation alters not only cell morphology (represented here by $R_{\textrm{U}}={10}\,\upmu \hbox {m}$ and $R_{\textrm{O}}={13}\,\upmu \hbox {m}$) but also membrane composition, cytoplasmic conductivity, and nuclear-to-cytoplasmic volume ratio all of which modify the impedance fingerprint captured by the coplanar sensing electrodes.

For each population, a single hMSC was trapped in the sensing zone between the impedance-sensor electrode pair ($W \times L={600}\,\upmu \hbox {m}\times {600}\,\upmu \hbox {m}$, $d={200}\,\upmu \hbox {m}$). A pulsed electroporation voltage was first applied at one of six amplitudes ($V_{\textrm{app}}=1$, 5, 10, 15, 20, and ${25}\,\text {V}$, corresponding to electric field strengths of approximately 50, 250, 500, 750, 1000, and ${1250}\,\hbox {V cm}^{-1}$ across the ${200}\,\upmu \hbox {m}$-wide fabricated inter-electrode gap). Immediately after pulsing, the function generator was switched to a low-amplitude (100 mV) sinusoidal signal swept from ${1}\,\hbox {kHz}$ to ${1}\,\hbox {MHz}$, and the complex impedance $Z = R_s + jX_s$ was recorded by the oscilloscope and transferred to the computer. Three impedance parameters were extracted at each frequency: the impedance magnitude |*Z*|, the series resistance $R_s$ (real part), and the series reactance $X_s$ (imaginary part). The following subsections present and interpret each spectral quantity. All values cited in the text are means computed from five independent experiments per cell type and voltage condition; the corresponding standard deviations are reported in the supplementary data tables.

### Impedance magnitude spectra

**Fig. 15:**
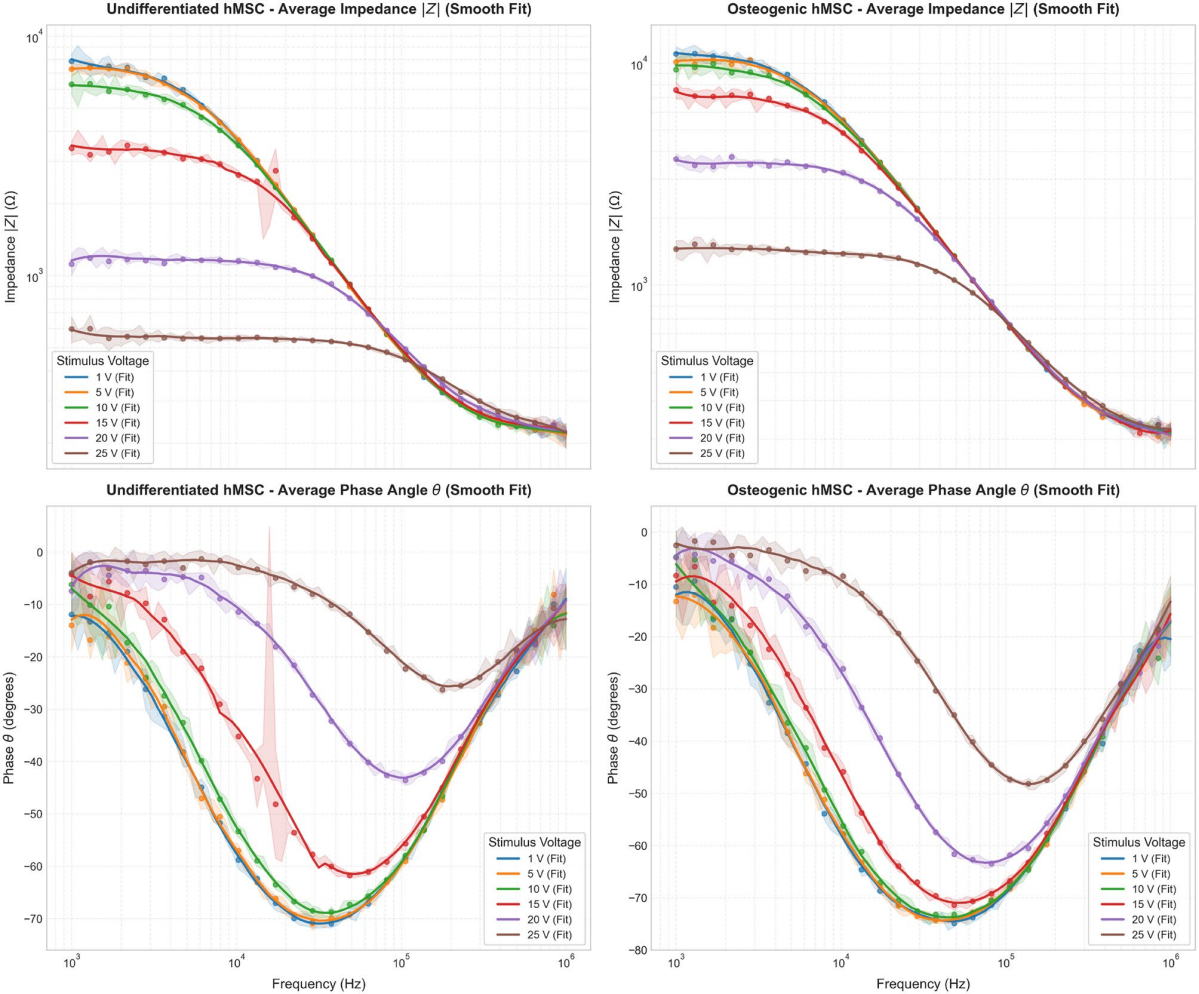
Average impedance magnitude (|*Z*|) and phase angle (*θ*) spectra of individual hMSCs measured over the frequency range of ${1}\,\hbox {kHz}$–${1}\,\hbox {MHz}$ following electroporation at six applied pulse voltages (1, 5, 10, 15, 20, and 25 V). Each cell-type/voltage condition was measured in five independent experimental runs; the plotted curves represent the average response across these runs, with smoothing applied only for visualization to reduce point-to-point noise and improve the visibility of voltage-dependent and cell-type-dependent differences. The upper row presents the impedance magnitude (|*Z*|), while the lower row shows the corresponding phase angle (*θ*). The left column corresponds to undifferentiated hMSCs, whereas the right column represents osteogenic-committed hMSCs. At low excitation voltages (1–5 V), both cell populations exhibit the characteristic *β*-dispersion behavior of intact cell membranes, with high low-frequency impedance and strongly negative phase angles resulting from membrane capacitive polarization. Increasing the electroporation voltage from 10 to 25 V progressively reduces the low-frequency impedance and shifts the phase response toward less capacitive behavior, indicating increased membrane permeabilization and the formation of conductive electroporation pores. Osteogenic-committed hMSCs consistently exhibit higher impedance magnitude and slightly larger phase lag than undifferentiated cells over most of the frequency range, reflecting differentiation-associated changes in cellular electrical properties. The greatest voltage-dependent differences are observed in the intermediate frequency region (approximately ${10}\,\hbox {kHz}$–${200}\,\hbox {kHz}$), where membrane polarization dominates the electrical response. Above approximately 500 kHz, both the impedance magnitude and phase responses converge for all applied voltages, indicating that the measured electrical behavior becomes increasingly governed by intracellular and bulk conductive pathways rather than membrane capacitance. These frequency-dependent responses demonstrate the sensitivity of swept-frequency impedance spectroscopy for monitoring electroporation-induced membrane changes and distinguishing undifferentiated from osteogenic-committed hMSCs.

Figure 15 displays the impedance magnitude |*Z*| as a function of frequency for undifferentiated hMSCs (left panel) and osteogenic hMSCs (right panel), measured after electroporation pulses of increasing amplitude.

Several observations connect these spectra directly to the simulation results of “Results and discussion”:

1. Voltage-dependent impedance reduction. The monotonic decrease in low-frequency |*Z*| with increasing applied voltage mirrors the monotonic increase in dynamic membrane conductivity $\sigma _m(t)$ predicted by the pore-density evolution model (Eq. (4) and Figs. 11, 12). At ${1}\,\text {V}$, the mean |*Z*| at ${1}\,\hbox {kHz}$ is ${7863}\,\Omega$ (undifferentiated) and ${11\,029}\,\Omega$ (osteogenic); by ${25}\,\text {V}$ these values fall to ${599}\,\Omega$ and ${1448}\,\Omega$, respectively a reduction of 92.4% and 86.9%.
2. Dispersion-frequency shift. At sub-threshold voltages (1 V to 5 V), the *β*-dispersion transition occurs near ${50}\,\hbox {kHz}$ for undifferentiated cells and near ${10}\,\hbox {kHz}$ for osteogenic cells. As the electroporation voltage increases, the effective membrane time constant $\tau _m = R_m C_m$ decreases (because $R_m$ drops due to pore formation while $C_m$ changes only modestly), shifting the dispersion frequency upward. This behavior is consistent with the charging-time descriptors $\tau _{\textrm{charge}}$ observed in the time-domain simulations for the two cell-type models and with the stronger response at higher field strengths (Table 5).
3. Differentiation-dependent baseline. The uniformly higher |*Z*| of osteogenic hMSCs at all voltages reflects the biophysical remodeling that accompanies osteogenic commitment: increased cell volume, enhanced extracellular matrix deposition, and altered membrane lipid composition all contribute to a higher effective impedance, providing a clear electrical signature for distinguishing differentiation states.

### Voltage-dependent impedance drop at the baseline crossover frequency

To condense the broadband impedance data into a single voltage-dependent metric, the percentage impedance drop at the fixed baseline crossover frequency $f_{c,0}$ is defined as:

7 $$\begin{aligned} \Delta |Z|_{f_{c,0}}(\%) = \frac{|Z|_{f_{c,0}}^{\,1\,\textrm{V}} - |Z|_{f_{c,0}}^{\,V_{\textrm{app}}}}{|Z|_{f_{c,0}}^{\,1\,\textrm{V}}} \times 100\;, \end{aligned}$$

where $|Z|_{f_{c,0}}^{\,1\,\textrm{V}}$ is the impedance magnitude at $f_{c,0}$ under the lowest (non-electroporating) reference voltage of ${1}\,\text {V}$, representing the intact-membrane state. The baseline crossover frequency $f_{c,0}$ is determined individually for each cell population from the inflection point of the |*Z*|-vs-frequency curve at ${1}\,\text {V}$, yielding $f_{c,0} = {5.6}\,\hbox {kHz}$ for undifferentiated hMSCs and $f_{c,0} = {5.8}\,\hbox {kHz}$ for osteogenic hMSCs. In the present work the percentage impedance drop metric is evaluated at 1 kHz rather than at $f_{c,0}$ directly, because the experimental 1 kHz values are tabulated with the highest repeatability (five replicates, supplementary Tables 6 and 8); the drop values at 1 kHz and at $f_{c,0}$ are consistent within measurement uncertainty. Evaluating the percentage drop at a fixed reference frequency $f_{c,0}$ avoids conflating baseline impedance differences with voltage-dependent frequency shifts, providing a physically clear metric for membrane permeabilization.

**Table 7:** Percentage impedance drop *Δ |Z|* at ${1}\,\hbox {kHz}$, referenced to the 1 V intact-membrane baseline for each cell population. Values are computed from the experimental mean |*Z*| at ${1}\,\hbox {kHz}$. The baseline crossover frequencies are $f_{c,0}={5.6}\,\hbox {kHz}$ for undifferentiated hMSCs and $f_{c,0}={5.8}\,\hbox {kHz}$ for osteogenic hMSCs.

$V_{\textrm{app}}$ (V)	Field (V/cm)	Undiff. drop (%)	Osteogenic drop (%)	Interpretation
1	50	0.0	0.0	Intact-membrane reference condition
5	250	7.4	8.2	Sub-threshold; marginal impedance reduction in both populations
10	500	19.9	15.1	Electroporation onset; stronger relative drop in undifferentiated hMSCs
15	750	56.7	31.4	Clear pore-mediated impedance reduction; undifferentiated hMSCs more strongly affected
20	1000	85.8	66.5	Established electroporation with strong conductance shunting
25	1250	92.4	86.9	Heavy electroporation; both populations show large impedance drop

Table 7 reveals several features that corroborate the computational predictions:

- Threshold behavior. Both curves remain near zero for $V_{\textrm{app}} \le {5}\,\text {V}$ ($E \le {250}\,\hbox {V cm}^{-1}$), indicating that the transmembrane potential induced at these low field strengths does not exceed the electroporation threshold ($V_{\textrm{th}} \approx {0.2}\,\text {V}$ to ${1}\,\text {V}$), consistent with Eq. (2). The 7.4% (undifferentiated) and 8.2% (osteogenic) drops observed at 5 V are within measurement variability and consistent with the sub-threshold regime.
- Monotonic, nonlinear increase. The sigmoidal shape of the *Δ |Z|*-vs-$V_{\textrm{app}}$ curve reflects the exponential dependence of pore density on $(V_{\textrm{TM}}/V_{\textrm{ep}})^2$ in Eq. (4): once $V_m$ exceeds $V_{\textrm{ep}}$, pore formation accelerates rapidly, producing a steep impedance drop.
- Differentiation-dependent susceptibility. Undifferentiated hMSCs exhibit a consistently larger impedance drop than osteogenic cells at every voltage above the threshold. At ${15}\,\text {V}$ the drops are 56.7% vs. 31.4%; at ${20}\,\text {V}$ they reach 85.8% vs. 66.5%. This finding is consistent with the broader model prediction that electroporation-induced membrane conductance changes can strongly reshape impedance spectra.
- Crossover-frequency difference. The slight difference in the baseline crossover frequency $f_{c,0}$ (${5.6}\,\hbox {kHz}$ vs. ${5.8}\,\hbox {kHz}$) reflects the distinct membrane capacitance-to-conductance ratios of the two populations, further enriching the feature set available for classification.

### Series reactance spectra

The series reactance $X_s$ (imaginary part of the complex impedance) captures the capacitive character of the cell membrane. The numerical differences in |*Z*|, $R_s$, $X_s$, and phase are reported in Tables 8 and 10.

The physical interpretation of the $X_s$ spectra is as follows. The cell membrane, modeled as a distributed parallel RC element (Fig. 2), presents a predominantly capacitive impedance at frequencies below the *β*-dispersion corner. The series reactance at these frequencies is therefore strongly negative (capacitive), with a magnitude proportional to the effective membrane capacitance $C_m = \varepsilon _m / d_m$. When electroporation creates pores, the membrane conductance $G_m$ increases dramatically (as quantified by $\sigma _m(t)$ in Figs. 11, 12), effectively placing a low-value resistance in parallel with $C_m$. This parallel resistance shunts the capacitive current at low frequencies, reducing the magnitude of $X_s$ and collapsing the trough.

Two key discriminative observations emerge from the data:

1. The absolute peak reactance $|X_{s,\textrm{pk}}|$ at sub-threshold voltages differs between the two populations (${1609}\,\Omega$ for undifferentiated vs. ${1921}\,\Omega$ for osteogenic at ${1}\,\text {V}$, ${1}\,\hbox {kHz}$), providing a baseline feature that distinguishes undifferentiated from osteogenic hMSCs even before electroporation is applied.
2. The rate at which $|X_{s,\textrm{pk}}|$ decreases with increasing voltage is cell-type-specific: the undifferentiated population exhibits strong trough attenuation by ${20}\,\text {V}$ (reducing to ${142}\,\Omega$ at ${1}\,\hbox {kHz}$), whereas the osteogenic population retains residual capacitive reactance at the same voltage (${310}\,\Omega$), suggesting a higher electroporation threshold or slower pore-formation kinetics in the differentiated state.

### Series resistance spectra

The series resistance $R_s$ (real part of *Z*) reflects the combined ohmic losses in the medium, the cytoplasm, and the electroporation-induced pore conductance. Table 8 summarizes the extracted low-frequency and high-frequency resistance values for both cell populations at six applied voltages.

**Table 8:** Series resistance $R_s$ summary extracted from the swept-frequency EIS measurements. Low-frequency values are the experimental means at ${1}\,\hbox {kHz}$. High-frequency values are the experimental means at ${1}\,\hbox {MHz}$, where the membrane capacitance is fully bypassed and $R_s$ converges to the solution/electrode limit. U = undifferentiated; O = osteogenic.

$V_{\textrm{app}}$ (V)	Field (V/cm)	$R_{s,1\,\textrm{kHz}}$ U (*Ω*)	$R_{s,1\,\textrm{kHz}}$ O (*Ω*)	High-*f* limit (*Ω*)	Interpretation
1	50	7687	10804	200–205	Intact membrane; osteogenic cells show higher low-frequency path resistance
5	250	7036	9819	213–205	Sub-threshold; $R_s$ remains near baseline in both populations
10	500	6235	9298	217–209	Electroporation onset; undifferentiated $R_s$ begins to fall
15	750	3390	7467	217–207	Pore-mediated shunting accelerates; growing inter-population contrast
20	1000	1106	3678	219–208	Strong transmembrane shunting, especially in undifferentiated hMSCs
25	1250	598	1445	207–215	Heavy electroporation; low-frequency $R_s$ approaching solution-dominated limit

The $R_s$ spectra provide complementary information to the |*Z*| and $X_s$ data:

- Low-frequency resistance as a pore-density proxy. At frequencies well below the *β*-dispersion corner, the membrane capacitance presents essentially infinite impedance, so the measured $R_s$ is dominated by the membrane resistance $R_m = d_m / \sigma _m$. As electroporation increases $\sigma _m$ (Figs. 11, 12), $R_m$ drops, and the low-frequency $R_s$ decreases accordingly. The undifferentiated $R_s$ falls from ${7687}\,\Omega$ at ${1}\,\text {V}$ to ${598}\,\Omega$ at ${25}\,\text {V}$; the osteogenic $R_s$ falls from ${10\,804}\,\Omega$ to ${1445}\,\Omega$ over the same range.
- High-frequency convergence. The convergence of all $R_s$ curves at high frequencies (to 200–219 *Ω* at ${1}\,\hbox {MHz}$ for both populations) confirms that the sensing electrodes are measuring the medium resistance at frequencies where displacement current bypasses the membrane entirely, as described by the second (capacitive) term in Eq. (3).
- Differentiation-dependent contrast. The baseline difference in low-frequency $R_s$ between undifferentiated and osteogenic hMSCs (${7687}\,\Omega$ vs. ${10\,804}\,\Omega$ at ${1}\,\text {V}$) constitutes a robust discriminative feature: osteogenic commitment enlarges the cell and alters membrane lipid composition, both of which increase the effective membrane resistance sensed by the coplanar electrodes.

### Consolidated impedance feature comparison

Table 9 consolidates the principal impedance features extracted from the experimental spectra for both stem-cell populations.

**Table 9:** Experimentally measured impedance features for undifferentiated and osteogenic hMSCs at selected applied voltages. $f_{c,0}$: baseline crossover frequency; $|Z|_{1\,\textrm{kHz}}$: impedance magnitude at ${1}\,\hbox {kHz}$; $R_{s,1\,\textrm{kHz}}$: series resistance at ${1}\,\hbox {kHz}$; $|X_{s,\textrm{pk}}|$: absolute peak series reactance across all measured frequencies; $\Delta |Z|_{1\,\textrm{kHz}}$: percentage impedance drop at ${1}\,\hbox {kHz}$ relative to the 1 V baseline.

Feature	Undifferentiated		Osteogenic		Unit
	1 V	25 V	1 V	25 V	
$f_{c,0}$	5.6		5.8		kHz
$|Z|_{1\,\textrm{kHz}}$	7 863	599	11 029	1 448	*Ω*
$R_{s,1\,\textrm{kHz}}$	7 687	598	10 804	1 445	*Ω*
$|X_{s,\textrm{pk}}|$	1 609	164	1 921	514	*Ω*
$\Delta |Z|_{1\,\textrm{kHz}}$	0	92.4	0	86.9	%

The consolidated data show that every impedance feature exhibits a measurable and systematic difference between the two stem-cell populations across the full range of applied voltages. Importantly, the experimental impedance features and the computational electroporation features are linked through a common physical mechanism: the formation of hydrophilic pores increases the membrane conductivity $\sigma _m$, which simultaneously (i) reduces the charging time $\tau _{\textrm{charge}}$ and alters the clamped voltage $V_{\textrm{clamp}}$ in the time domain, and (ii) decreases |*Z*|, $R_s$, and $|X_s|$ in the frequency domain. This unified interpretation supports the use of the hybrid analytical–numerical model as an interpretive tool for designing impedance-based stem-cell classification protocols on the microfluidic platform.

### Biophysical origin of impedance differences: cell size vs. membrane composition

To address the biophysical mechanisms underlying the observed electrical differences between undifferentiated and osteogenic hMSCs, we must distinguish between the contributions of cell size and differentiation-induced membrane composition changes. Both factors play distinct, coupled roles in the resulting impedance spectra:

1. Sub-threshold regime (1 V and 5 V): in the unpermeabilized state, the cell membrane behaves as a high-resistivity barrier ($R_m \approx {0.01}\,\Omega \,\hbox {m}^{2}$ from the $G_m d_m$ parameterization). Here, osteogenic-committed hMSCs show higher baseline impedance magnitude ($|Z| \approx {11\,029}\,\Omega$ vs. $\approx {7863}\,\Omega$ for undifferentiated cells at ${1}\,\hbox {kHz}$, ${1}\,\text {V}$) and higher series resistance ($R_s \approx {10\,804}\,\Omega$ vs. $\approx {7687}\,\Omega$). These differences are consistent with the larger representative osteogenic geometry ($R_{\textrm{O}}\approx {13}\,\upmu \hbox {m}$ vs. $R_{\textrm{U}}\approx {10}\,\upmu \hbox {m}$), together with differentiation-induced changes in morphology and membrane properties. The larger apparent volume of osteogenic cells displaces more conductive medium within the sensing region, creating a stronger Coulter-like current occlusion. Additionally, the larger effective surface area contributes to the deeper series reactance peak ($|X_{s,\textrm{pk}}| \approx {1921}\,\Omega$ vs. ${1609}\,\Omega$ at ${1}\,\hbox {kHz}$, ${1}\,\text {V}$). These baseline features are therefore influenced by both representative cell geometry and differentiation-induced changes in membrane folding, effective surface area, and cytoplasm electrical properties.
2. Supra-threshold regime (15 V to 25 V): once the applied voltage exceeds the apparent electroporation threshold, the electrical contrast changes: undifferentiated hMSCs exhibit a lower low-frequency impedance magnitude and resistance ($R_s \approx {3390}\,\Omega$ at 15 V) than their osteogenic counterparts ($R_s \approx {7467}\,\Omega$). In a passive Schwan-type picture, larger cells would be expected to experience a higher induced transmembrane potential under a uniform field. The experimental observation that osteogenic-committed hMSCs retain a larger impedance at high voltage therefore suggests that factors beyond cell radius, such as differentiation-dependent membrane composition, cytoskeletal state, surface morphology, and extracellular matrix deposition, may influence the apparent electroporation response. These factors were not explicitly parameterized in the radius-parameterized cell-type model, so the interpretation is framed as a plausible biophysical explanation rather than a uniquely proven mechanism.

This analysis indicates that baseline (sub-threshold) impedance features are strongly influenced by size-related current occlusion and capacitance scaling, whereas voltage-dependent trajectories may also reflect differentiation-related membrane and surface properties. The integration of both sub-threshold and supra-threshold features in the combined feature space ${\mathcal {F}}_{\textrm{combined}}$ is therefore proposed as a useful basis for future classification studies, pending validation on larger biological datasets.

Furthermore, the experimental observation that osteogenic hMSCs require higher applied voltages to reach the same percentage impedance drop as undifferentiated hMSCs (Table 7) is consistent with the hypothesis that differentiation alters the effective membrane and surface response to pulsed fields. Taken together, the time-domain simulation features and the frequency-domain impedance measurements form a complementary candidate feature set ${\mathcal {F}}_{\textrm{combined}} = \{\tau _{\textrm{charge}},\; V_{\textrm{clamp}},\; N_{\max },\; r_{p,\max },\; \sigma _{m,\max },\; f_{c,0},\; \Delta |Z|_{f_{c,0}},\; R_{s,1\,\textrm{kHz}},\; |X_{s,\textrm{pk}}|\}$ for future machine-learning studies of label-free stem-cell differentiation-state identification within the microfluidic electroporation platform.

### Per-voltage impedance comparison between undifferentiated and osteogenic hMSCs

“Impedance magnitude spectra”, “Voltage-dependent impedance drop at the baseline crossover frequency”, “Series reactance spectra” and “Series resistance spectra” presented the impedance spectra grouped by cell type and overlaid for all six applied voltages, highlighting the voltage-dependent evolution within each population. A complementary and equally informative representation is to fix the applied voltage and overlay the two populations in a numerical comparison. Table 10 adopts this perspective: each row corresponds to a single electroporation voltage and reports the impedance magnitude |*Z*|, series resistance $R_s$, and peak reactance magnitude $|X_{s,\textrm{pk}}|$ for undifferentiated and osteogenic hMSCs. This table directly reports the *inter-population contrast* available at each operating point of the electroporation pulse generator.

#### Sub-threshold regime (1 V / 50 V $\hbox {cm}^{-1}$ and 5 V / 250 V $\hbox {cm}^{-1}$)

The first two rows of Table 10 correspond to applied voltages of ${1}\,\text {V}$ and ${5}\,\text {V}$, respectively. Several consistent features are observed:

1. Impedance magnitude (|*Z*|). Osteogenic hMSCs exhibit a uniformly higher |*Z*| than undifferentiated cells across the entire frequency range. At ${1}\,\hbox {kHz}$ the separation is approximately $|Z|_{\textrm{osteo}} \approx {11\,029}\,\Omega$ vs. $|Z|_{\textrm{undiff}} \approx {7863}\,\Omega$ a factor of approximately 1.4. This baseline difference originates from the biophysical remodeling that accompanies osteogenic commitment: an increase in cell volume, membrane surface area, and extracellular matrix thickness, all of which raise the effective capacitive impedance in the cell–electrode system modelled by the distributed RC boundary condition (Eq. (3)). The characteristic *β*-dispersion roll-off is clearly visible in both curves; however, it begins at a lower frequency for the osteogenic population ($\sim \!{3}\,\hbox {kHz}$) than for the undifferentiated population ($\sim \!{10}\,\hbox {kHz}$), reflecting the larger membrane time constant $\tau _m = R_m C_m$ associated with the bigger cell. Above $\sim \!{500}\,\hbox {kHz}$ the two curves converge to a common asymptote ($|Z| \approx {250}\,\Omega$), which is set by the experimental PBS conductivity and electrode geometry.
2. Series resistance ($R_s$). The osteogenic population presents a markedly higher low-frequency $R_s$ ($\approx {10\,804}\,\Omega$) compared with the undifferentiated population ($\approx {7687}\,\Omega$). Because the membrane is intact at these sub-threshold voltages, the low-frequency current is forced to flow exclusively through the narrow extracellular sheath around the cell. Both curves converge to $R_s \approx 200$–*215~Ω* at ${1}\,\hbox {MHz}$.
3. Series reactance ($X_s$). Both populations display a pronounced negative (capacitive) trough. For the undifferentiated hMSCs the trough minimum is $|X_{s,\textrm{pk}}| \approx {1609}\,\Omega$ at ${1}\,\hbox {kHz}$; for the osteogenic hMSCs it is $\approx {1921}\,\Omega$, reflecting the larger total membrane capacitance of the bigger cell.
4. Invariance between 1 and 5 V. The tabulated features at ${5}\,\text {V}$ remain very close to those at ${1}\,\text {V}$ for both cell types. This invariance confirms that the transmembrane potential induced by $E \le {250}\,\hbox {V cm}^{-1}$ remains below the electroporation threshold, so no pores are formed and the membrane retains its native high resistivity.

Even without electroporation, the impedance spectra at sub-threshold voltages already provide three robust features low-frequency |*Z*|, low-frequency $R_s$, and $|X_{s,\textrm{pk}}|$ that clearly separate undifferentiated from osteogenic hMSCs.

#### Electroporation onset (10 V / 500 V $\hbox {cm}^{-1}$)

The ${10}\,\text {V}$ row of Table 10 captures the transition from the sub-threshold regime to the onset of electroporation at 10 V (${500}\,\hbox {V cm}^{-1}$).

- |*Z*|. The mean undifferentiated $|Z|_{1\,\textrm{kHz}}$ has fallen to ${6300}\,\Omega$ (from ${7863}\,\Omega$ at 1 V), whereas the osteogenic value remains at $\approx {9365}\,\Omega$. The corresponding impedance drops are 19.9% and 15.1%, respectively (Table 7).
- $R_s$. The undifferentiated $R_{s,1\,\textrm{kHz}}$ has decreased from ${7687}\,\Omega$ to ${6235}\,\Omega$, consistent with the emergence of pore conductance. The osteogenic value decreases more modestly, from ${10\,804}\,\Omega$ to ${9298}\,\Omega$.
- $X_s$. The reactance trough of the undifferentiated population has shallowed (from ${1609}\,\Omega$ to ${641}\,\Omega$ at ${1}\,\hbox {kHz}$), indicating that newly formed pores are beginning to shunt the membrane capacitance. The osteogenic trough remains deeper at ${796}\,\Omega$.

#### Established electroporation (15 V / 750 V $\hbox {cm}^{-1}$)

At ${15}\,\text {V}$ (${750}\,\hbox {V cm}^{-1}$), electroporation is active in both cell types. A striking qualitative change emerges:

1. Contrast inversion in |*Z*| and $R_s$. At sub-threshold voltages, the osteogenic population always exhibited higher |*Z*| and $R_s$ than the undifferentiated population. At 15 V, however, the undifferentiated cells now show *lower* low-frequency |*Z*| ($\approx {3409}\,\Omega$) and $R_s$ ($\approx {3390}\,\Omega$) than the osteogenic cells ($|Z| \approx {7563}\,\Omega$, $R_s \approx {7467}\,\Omega$). The impedance-drop metric at this voltage (Table 7) reads $\Delta |Z|_{1\,\textrm{kHz}} \approx 56.7\%$ (undifferentiated) versus $\approx 31.4\%$ (osteogenic).
2. Reduced but still measurable $X_s$ contrast. The undifferentiated reactance has collapsed to $|X_{s,\textrm{pk}}| \approx {249}\,\Omega$ (1 kHz), whereas the osteogenic value remains at $\approx {1102}\,\Omega$ nearly *4.4×* larger, providing strong capacitive-reactance-based discrimination even during active electroporation.
3. High-frequency convergence. Above $\sim \!{200}\,\hbox {kHz}$, both |*Z*| curves merge and all $R_s$ curves approach the common medium resistance.

#### Heavy electroporation (20 V / 1000 V $\hbox {cm}^{-1}$ and 25 V / 1250 V $\hbox {cm}^{-1}$)

At 20 V:

- The undifferentiated $|Z|_{1\,\textrm{kHz}}$ is now $\approx {1119}\,\Omega$, and $R_s$ is correspondingly $\approx {1106}\,\Omega$, indicating that the membrane is so heavily perforated that it no longer presents a significant barrier to ionic current.
- The osteogenic $|Z|_{1\,\textrm{kHz}}$ still shows a clear plateau at $\approx {3696}\,\Omega$ and $R_s \approx {3678}\,\Omega$, indicating that a portion of the osteogenic membrane remains intact.
- The undifferentiated $|X_{s,\textrm{pk}}|$ has nearly vanished (${142}\,\Omega$ at ${1}\,\hbox {kHz}$), while the osteogenic peak remains at ${310}\,\Omega$.
- The impedance-drop metric (Table 7) reaches $\approx 85.8\%$ (undifferentiated) and $\approx 66.5\%$ (osteogenic).

At $${25}\,\text {V}$$:

- Both populations are now heavily electroporated. The undifferentiated $|Z|_{1\,\textrm{kHz}}$ reaches ${599}\,\Omega$ and $R_s \approx {598}\,\Omega$, with an $|X_{s,\textrm{pk}}|$ of only ${41}\,\Omega$ nearly electrically transparent.
- The osteogenic population still exhibits $|Z|_{1\,\textrm{kHz}} \approx {1448}\,\Omega$ and $R_s \approx {1445}\,\Omega$ about *2.4×* higher than the undifferentiated value, demonstrating that inter-population contrast persists even under the most extreme pulsing conditions. The osteogenic $|X_{s,\textrm{pk}}|$ at ${1}\,\hbox {kHz}$ is ${64}\,\Omega$, and the peak absolute reactance across all frequencies is ${514}\,\Omega$ (at ${100}\,\hbox {kHz}$), in contrast to ${164}\,\Omega$ for undifferentiated hMSCs.
- Table 7 reports $\Delta |Z|_{1\,\textrm{kHz}} \approx 92.4\%$ (undifferentiated) and $\approx 86.9\%$ (osteogenic) at ${25}\,\text {V}$ approaching the theoretical maximum of 100%, which would correspond to total removal of the cell from the sensing zone.

#### Consolidated per-voltage analysis

Table 10 summarises the key impedance features extracted from the per-voltage impedance comparisons and quantifies the inter-population contrast at each voltage.

**Table 10:** Selected impedance features for undifferentiated (U) and osteogenic (O) hMSCs at each applied voltage, together with the inter-population contrast $\Delta _{\mathrm {U{-}O}} = |\text {feature}_{\textrm{O}} - \text {feature}_{\textrm{U}}|$. $|Z|_{1\,\textrm{kHz}}$ and $R_{s,1\,\textrm{kHz}}$ are the experimental means (in *Ω*). $|X_{s,\textrm{pk}}|$ is the largest absolute mean reactance observed across the three measurement frequencies (in *Ω*).

$V_{\textrm{app}}$	$|Z|_{1\,\textrm{kHz}}$		*Δ*	$R_{s,1\,\textrm{kHz}}$		*Δ*	$|X_{s,\textrm{pk}}|$		*Δ*
(V)	U	O		U	O		U	O	
1	7863	11,029	3166	7687	10,804	3117	1609	1921	312
5	7282	10,126	2844	7036	9819	2783	1776	2355	579
10	6300	9365	3065	6235	9298	3063	641	796	155
15	3409	7563	4154	3390	7467	4077	417	1102	685
20	1119	3696	2577	1106	3678	2572	356	644	288
25	599	1448	849	598	1445	847	164	514	350

The per-voltage comparison reveals four overarching trends that are interpreted using the electroporation physics implemented in the computational model:

1. Persistent inter-population contrast at every voltage. At no voltage from ${1}\,\text {V}$ (intact membranes) to ${25}\,\text {V}$ (heavily disrupted membranes) do the two populations become indistinguishable.
2. Contrast inversion above the threshold. Below the electroporation threshold ($\le {5}\,\text {V}$), osteogenic hMSCs always present higher |*Z*| and $R_s$ than undifferentiated hMSCs. Above the threshold ($\ge {15}\,\text {V}$), the undifferentiated population drops below the osteogenic.
3. Reactance contrast peaks near electroporation onset. The inter-population $|X_{s,\textrm{pk}}|$ contrast reaches ${685}\,\Omega$ at 15 V, even though the absolute trough depths are smaller than at sub-threshold voltages.
4. Dispersion-frequency shift encodes pore state. The *β*-dispersion corner frequency shifts upward with increasing voltage and differs between the two populations, providing an additional candidate feature that reports on the instantaneous membrane conductivity.

The per-voltage comparison data close the loop between the model predictions and the experimental measurements:

- The model predicts that $\sigma _m(t)$ increases with applied field strength. The per-voltage impedance data provide the frequency-domain counterpart: each increment in $\sigma _m$ manifests as a quantifiable reduction in |*Z*|, $R_s$, and $|X_s|$ at frequencies below $f_\beta$.
- The model uses two cell-type representations ($R_{\textrm{U}}={10}\,\upmu \hbox {m}$ and $R_{\textrm{O}}={13}\,\upmu \hbox {m}$). The experimental per-voltage spectra show that the impedance signatures of the two measured populations are quantitatively distinct at every tested voltage, supporting the proposed link between model features ($\tau _{\textrm{charge}}$, $V_{\textrm{clamp}}$, $N_{\max }$, and $\sigma _{m,\max }$) and measurable impedance differences.
- The combined feature set provides a rich, multi-dimensional descriptor ${\mathcal {F}} = \{|Z|_{1\,\textrm{kHz}},\; R_{s,1\,\textrm{kHz}},\; |X_{s,\textrm{pk}}|,\; f_{\beta },\; \Delta |Z|_{f_{c,0}}\}$ at each applied voltage. When augmented with the time-domain features from the computational model, this descriptor provides a physically grounded basis for future evaluation of stem-cell differentiation states.

## Conclusion

This study presented a hybrid analytical–numerical framework for interpreting electroporation dynamics and swept-frequency impedance spectroscopy in hMSC-focused microfluidic experiments. The model resolves transmembrane potential, pore density, pore radius, and membrane conductivity for undifferentiated and osteogenic hMSC representations, while the impedance measurements provide complementary frequency-domain evidence of voltage-dependent membrane permeabilization in the same two biological populations.

The revised interpretation separates passive pre-poration scaling from post-threshold voltage clamping. Before permeabilization, the Schwan-type relation predicts that the induced transmembrane potential scales with cell radius. After pore formation, however, pore-mediated membrane conductance limits further voltage buildup, leading to clamped polar voltages of about 1.3 V to 1.4 V under the selected supra-threshold simulation conditions. Therefore, small differences between clamped voltages or charging times are not treated as validated discriminative biomarkers. Pore density, pore-radius evolution, and membrane conductivity are instead interpreted as model-derived descriptors that support mechanistic comparison with the impedance spectra.

The impedance experiments show voltage-dependent reductions in |*Z*|, $R_s$, $|X_s|$, and phase-angle magnitude, consistent with increased membrane conductance during electroporation. The simulation and experiment therefore agree through the same physical pathway: the model predicts that increasing electric-field exposure raises pore density, expands nanometre-scale pore radii, and increases the effective membrane conductivity $\sigma _m$; experimentally, this pore-mediated conductance appears as a progressive decrease in low-frequency impedance magnitude, a collapse of the series-resistance plateau, attenuation of the capacitive reactance trough, and a phase response that shifts toward less capacitive behavior. The stronger high-voltage impedance drop observed in undifferentiated hMSCs (92.4 % at 25 V) compared with osteogenic hMSCs (86.9 %) is consistent with the model-derived interpretation that the two cell states have different effective membrane responses during electroporation. Calibrated microbead measurements using 10.4 *μ*m and 24.9 *μ*m polystyrene beads provide a cell-free calibration of size-dependent impedance contrast in the same sensing geometry. The model–data comparison is framed as mechanistic consistency rather than one-to-one quantitative validation of every simulated state variable.

The combined feature space,

$${\mathcal {F}}_{\textrm{combined}} = \bigl \{ \tau _{\textrm{charge}},\; V_{\textrm{clamp}},\; N_{\max },\; r_p(t),\; \sigma _{m,\max },\; f_c,\; \Delta |Z|_{f_{c,0}},\; R_{s,1\,\textrm{kHz}},\; |X_{s,\textrm{pk}}| \bigr \},$$

provides a physically interpretable candidate descriptor for future label-free characterization. The current study does not present a trained classifier, cross-validation, external test set, direct pore imaging, or viability/delivery-efficiency assays; therefore, the proposed feature space should be regarded as a foundation for future validation rather than a completed classification or cell-sorting method.

### Limitations

The present work should be interpreted primarily as an engineering study focused on the development and validation of an analytical numerical electroporation model integrated with swept-frequency impedance spectroscopy for label-free stem-cell characterization. Accordingly, the computational framework employs literature-reported geometric and dielectric properties representative of undifferentiated and osteogenic-committed human mesenchymal stem cells to establish physiologically relevant model parameters.

The computational model employs a simplified single-shell spherical representation of the cell membrane and cytoplasm and does not explicitly account for the nucleus, intracellular organelles, non-spherical morphology, membrane heterogeneity, cytoskeletal remodeling, or extracellular matrix interactions. Pore density and pore radius are inferred from established electroporation kinetics rather than directly visualized experimentally. Although the impedance measurements provide experimental support for the proposed framework, the simulation and experimental electrical conditions are not identical; therefore, the experimental results provide mechanistic validation rather than complete quantitative validation of every modeled state variable. In addition, the impedance spectra were obtained from repeated measurements and averaged responses, improving measurement robustness while potentially masking biological heterogeneity among individual cells.

The biological component of this work was intended to demonstrate the feasibility of the proposed impedance sensing methodology rather than to provide a comprehensive biological characterization of stem-cell differentiation. Consequently, biological properties were incorporated into the engineering model using well-established values reported in the literature and standard osteogenic differentiation protocols. Future studies will further strengthen the biological interpretation by incorporating additional biological characterization alongside the proposed engineering framework.

## Supplementary Information

Supplementary Information.

## Data Availability

All data generated or analysed during this study are included in this published article and its supplementary information files. The raw swept-frequency impedance spectroscopy datasets for undifferentiated and osteogenic-committed human mesenchymal stem cells (hMSCs) are provided as Supplementary Data files. The supplementary files also include the algorithmic steps and Python implementation used for the COMSOL-based electroporation modelling.
