# Supplementary material for: Hybrid modeling of electroporation and impedance spectroscopy for label free characterization of stem cells
Source: Sci Rep. 2026 Jul 23;16:23090. doi: 10.1038/s41598-026-62691-0 (PMC13396236; doi:10.1038/s41598-026-62691-0)
Supplement: Supplementary file 1 — Supplementary Information. [file 41598_2026_62691_MOESM1_ESM.docx]

Qualitative Optical Microscopy of Human Mesenchymal Stem Cells. Representative bright-field microscopy images acquired during the original experimental campaign are provided to illustrate the morphology and localization of the human mesenchymal stem cells (hMSCs) investigated in this study. The images include cells positioned within the sensing region of the impedance microelectrodes, representative viable cells with preserved morphology, and representative dead cells exhibiting structural deterioration. These microscopy observations are presented solely as qualitative supporting evidence for the experimental proof-of-concept and the operation of the proposed microfluidic impedance platform. They are not intended to serve as quantitative biological validation of osteogenic differentiation or cell viability, which require dedicated biochemical or fluorescence-based assays.

**
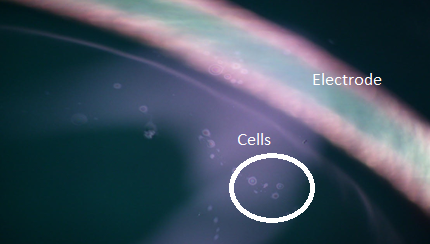
**

**Supplementary Figure S1: Representative optical microscopy image of cells captured within the sensing region of the microfluidic impedance platform.** The image illustrates human mesenchymal stem cells (hMSCs) positioned over the sensing electrodes during impedance measurements, demonstrating successful cell localization within the active sensing area. These observations provide qualitative proof-of-concept for the operation of the proposed impedance sensing platform and confirm the interaction of suspended cells with the electrode region during electrical characterization.

**
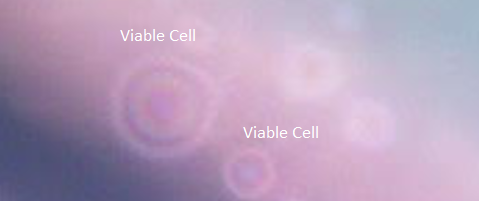
**

**Supplementary Figure S2: Representative optical microscopy image of viable human mesenchymal stem cells (hMSCs).** The cells exhibit preserved morphology with intact membrane boundaries and characteristic cellular appearance during the impedance measurement experiments. These images were acquired during the original experimental campaign and are presented as qualitative observations supporting the feasibility of the experimental platform. They are not intended as quantitative viability assessment or biological validation.


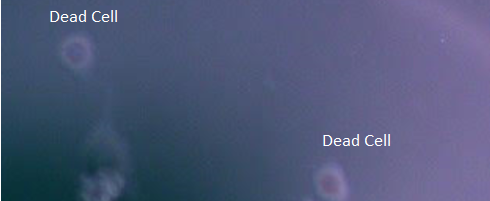


**Supplementary Figure S3: Representative optical microscopy image of dead human mesenchymal stem cells (hMSCs).** The image shows cells exhibiting morphological changes consistent with loss of membrane integrity and structural deterioration following irreversible cellular damage. These representative observations provide qualitative evidence of different cellular states encountered during the experiments and are included to complement the electrical impedance measurements. They should not be interpreted as quantitative cell viability analysis.
